# ‘Prehabilitation Should Adapt to the Person, not the Other Way Around’: A Qualitative Study Exploring Stakeholder Perceptions of Prehabilitation for Total Hip and Knee Arthroplasty

**DOI:** 10.1002/msc.70266

**Published:** 2026-10-08

**Authors:** N. Burgess, E. Walkley, S. Berney, L. Denehy, L. Edbrooke, M. Graco

**Affiliations:** ^1^ Department of Physiotherapy Melbourne School of Health Sciences University of Melbourne Melbourne Victoria Australia; ^2^ Department of Physiotherapy Austin Health Melbourne Victoria Australia; ^3^ Orthopaedic Research Centre at Austin (ORCA) Austin Health Melbourne Victoria Australia; ^4^ Department of Surgery Melbourne Medical School University of Melbourne Melbourne Victoria Australia; ^5^ Centre for Health Services Research in Cancer Peter MacCallum Cancer Centre Melbourne Victoria Australia; ^6^ Department of Anaesthesia, Perioperative Medicine and Pain Medicine Peter MacCallum Cancer Centre Melbourne Victoria Australia; ^7^ Sir Peter MacCallum Department of Oncology University of Melbourne Melbourne Victoria Australia; ^8^ Institute of Breathing and Sleep Austin Health Melbourne Victoria Australia

**Keywords:** acceptability, barriers and enablers, hip arthroplasty, knee arthroplasty, prehabilitation

## Abstract

**Background:**

Prehabilitation aims to increase functional capacity and physiological reserve prior to surgery, to improve post‐operative outcomes. Despite growing research on prehabilitation for total hip arthroplasty (THA) and total knee arthroplasty (TKA), its acceptability and barriers and enablers to implementation in clinical settings are not well understood.

**Objective:**

This study, involving patients and healthcare professionals (HCPs), aimed to explore the acceptability of prehabilitation, identify factors affecting prehabilitation access, uptake and delivery, and investigate perspectives on feasible and preferred care models, and prehabilitation content.

**Methods:**

Semi‐structured interviews were conducted with patients who had undergone or were awaiting THA or TKA and HCPs involved in their care at a public hospital in Melbourne, Australia. Interview guides were informed by the Theoretical Framework of Acceptability and the Theoretical Domains Framework. Data were analysed using an inductive‐deductive approach.

**Results:**

Thirty interviews were completed with patients (*n* = 15) and HCPs (*n* = 15). Three major concepts emerged: (1) *Understanding and beliefs*—while knowledge varied, prehabilitation was generally viewed as acceptable and beneficial; (2) *Barriers and enablers*—factors influencing uptake and delivery were identified at three levels of care; patient‐level (e.g., mindset), HCP‐level (e.g., confidence discussing prehabilitation with patients), and system‐level (e.g., long surgical waitlists making prehabilitation timing challenging); (3) *Strategies for success*—both groups emphasised tailored prehabilitation, multi‐modal delivery, and support throughout the surgical pathway as important intervention components.

**Conclusion:**

This study identified factors influencing acceptability, uptake, and delivery of prehabilitation at various care levels. The findings will inform the development of a flexible, stakeholder‐informed prehabilitation model to empower and optimise patients prior to THA and TKA.

## 1 Introduction

Total hip arthroplasty (THA) and total knee arthroplasty (TKA) are among the most common and cost‐effective elective surgeries performed globally (Daigle et al. 2012; Lan et al. 2020), improving pain, function and quality of life for individuals with end‐stage osteoarthritis (Ethgen et al. 2004). However, the pre‐operative period is often characterised by worsening pain, reduced physical function, and poorer health‐related quality of life (Abujaber et al. 2023; Salaffi et al. 2005). This pre‐operative deterioration has implications for post‐operative recovery, including increased length of hospital stay, delayed recovery of physical activities, and increased risk of complications (Elings et al. 2016; Hoogeboom et al. 2014; Raad et al. 2019). Preoperative rehabilitation, or ‘prehabilitation’, has been proposed to optimise preoperative function and prevent unsatisfactory postoperative outcomes after THA or TKA (Vaishya et al. 2024). Prehabilitation is a broad and variably defined concept. For the purposes of this study, prehabilitation was conceptualised as a preoperative intervention intended to prepare patients for surgery and recovery through optimisation of modifiable factors. Consistent with the broader prehabilitation literature, this may comprise multiple components, including exercise, education, nutritional and psychological support, delivered individually or in combination (Fleurent‐Grégoire et al. 2024).

The effectiveness of prehabilitation for patients undergoing THA or TKA remains uncertain, with methodological and intervention heterogeneity limiting the strength of conclusions that can be drawn from the existing evidence. Substantial variation in program content, duration, timing, and delivery makes it difficult to determine which components, or combinations of components, are most effective, while the high risk of bias across many trials and inconsistent selection of outcome measures further complicate interpretation and comparison of findings (Adebero et al. 2024; Punnoose et al. 2023; Vervullens et al. 2023). There are no universally adopted standards to guide the aims or content of prehabilitation programs, and the absence of a core outcome set has resulted in substantial heterogeneity in the measures used to evaluate effectiveness (Burgess et al. 2025).

Despite a growing body of randomised controlled trials evaluating orthopaedic prehabilitation, limited research has explored the barriers, facilitators and acceptability of these programs from the perspectives of patients and healthcare professionals (HCPs). This represents an important evidence gap as acceptability, and knowledge of barriers and enablers, are key determinants of intervention uptake and sustained implementation in clinical practice, and may not be captured by trials evaluating clinical effectiveness alone. Most qualitative work in the field has involved evaluating a prehabilitation intervention, rather than informing intervention development (Clode et al. 2018; Halsen et al. 2023; Husted et al. 2020). Recently published research priorities for THA and TKA prehabilitation (James Lind Alliance 2025) include exploring barriers to optimal prehabilitation delivery modes, and designing strategies to enhance engagement.

In the absence of clear recommendations on the ‘what, when and how’ of prehabilitation for this patient group, understanding patient and HCP perspectives is essential to maximise the acceptability, feasibility and effectiveness of prehabilitation interventions. This study aimed to:

1. Explore the acceptability of prehabilitation;
2. Investigate factors affecting prehabilitation access, uptake and delivery; and
3. Explore perspectives on preferred prehabilitation care models.

## 2 Methods

### 2.1 Study Design

A semi‐structured interview guide was developed a priori by two members of the research team (NB and MG) (Supporting Information S1: eTable 1), informed by the Theoretical Domains Framework (TDF) (Cane et al. 2012) and Theorical Framework of Acceptability (TFA) (Sekhon et al. 2017) (Supporting Information S1: eTables 2 and 3). The TDF is a synthesis of multiple theories on behaviour change. It is made up of 14 distinct domains designed to understand what factors influence HCP's behaviour, for example, knowledge of an intervention or social influences (Cane et al. 2012). The TFA assesses how acceptable healthcare interventions are based on people's cognitive and emotional responses, such as how someone feels about the intervention or the perceived amount of effort to participate. There were 7 domains in the original iteration of the framework; however, it has been updated to now include 8 domains (Sekhon et al. 2017; Paynter et al. 2023). When used in conjunction, the TDF and TFA facilitate the exploration of the acceptability of, and barriers and facilitators to, interventions such as prehabilitation. Questions in the interview guide were mapped to the relevant constructs within each framework to ensure comprehensive coverage of domains pertinent to the acceptability, uptake and delivery of prehabilitation. Feedback on the topic guide was then sought from other members of the research team and local clinical experts. Following the initial interviews, the interview guide was iteratively refined to enhance question clarity and optimise the structure and flow of the interview, while maintaining alignment with the underlying theoretical frameworks.

Research rigour was demonstrated by adherence to the Consolidated Criteria for Reporting Qualitative Research (COREQ) guidelines (Tong et al. 2007) (Supporting Information S1: eTable 4).

### 2.2 Participants and Setting

The study was based at a public hospital in Victoria, Australia, which performs over 400 THA and TKA annually. At the study site, patients awaiting THA or TKA were offered participation in a six‐week, physiotherapist‐delivered, group‐based exercise prehabilitation program. Participation was voluntary and required patients to opt in to the program. Ethical approval was sought through the hospital's Human Research Ethics Committee *(HREC/109358/2024).*

#### 2.2.1 Patient Group

Patients on the surgical waitlist for a THA or TKA or who had received a THA or TKA within the last 6 months were eligible, regardless of whether they had completed any form of prehabilitation. To include the perspectives of culturally and linguistically diverse participants, people who were unable to converse in English were offered to complete an interview with an interpreter.

#### 2.2.2 Healthcare Professional Group

HCPs providing care to patients undergoing a THA or TKA at the study site were eligible to participate. This included orthopaedic surgeons, anaesthetists, physiotherapists, and surgical liaison nurses.

### 2.3 Sample Size

This study aimed to recruit at least 15 patients and 15 HCPs. A purposive sampling technique was used to ensure diversity, with HCPs selected by profession and experience, and patients by surgery type, surgery status, cultural and linguistic diversity, and engagement with the site's prehabilitation service. Thematic analysis began after 12 interviews, and data collection continued until three consecutive interviews yielded no new themes, indicating saturation.

### 2.4 Data Collection and Analysis

Interviews were conducted online via Microsoft Teams or face‐to‐face (participant preference) by a single researcher (NB) and audio‐recorded. Interviews were initially transcribed using the built‐in transcription function available through Microsoft Teams. Using the original audio‐recordings, the transcription was then cross‐checked for accuracy and revised as required by one researcher (NB). Thematic analysis of qualitative data was conducted using an iterative inductive‐deductive approach informed by the Framework Analysis method (Gale et al. 2013). Following transcription, 20% of de‐identified transcripts were independently inductively coded by two researchers (NB, EW) to generate preliminary codes (Braun and Clarke 2006). A codebook was developed to standardise definitions, and regular meetings with a third qualitative researcher (MG) were held to develop and refine the coding framework. NB coded the remaining transcripts using this framework in NVivo 12 (https://lumivero.com/products/nvivo/), adding more codes as new themes emerged. The research team (NB, EW, MG) finalised the thematic structure through consensus. Sub‐themes and constituent codes were then mapped to the TDF and TFA by NB and MG. EW reviewed the mapping; discrepancies were resolved through discussion.

## 3 Results

Of the 47 people invited to participate in the study, 9 declined (*n* = 3 HCPs, *n* = 6 patients) and 8 HCPs did not respond. Reasons for declining included time constraints (*n* = 6), not interest (*n* = 2) and medically unwell (*n* = 1). Thirty participants completed interviews, including 15 HCPs and 15 patients; 19 were conducted online and 11 face‐to‐face; and three participants utilised interpreters as English was not their preferred language. Table 1 shows the demographics of the participants.

**TABLE 1:** Demographics.

Patient participants (*n* = 15)	*n* (%)
Gender	
Male	7 (47)
Female	8 (53)
Length of interview, mean [range], time	0:38:23 [00:14:09–1:02:01]
Age, mean ± SD, years	69.2 ± 7.9
Timepoint of contact	
Pre‐operative	6 (40)
Post‐operative	9 (60)
Joint affected	
Knee	7 (47)
Hip	8 (53)
Days on waitlist^a^, median [IQR]	527 [169–749]
Completed prehabilitation	
Yes	7 (47)
No	8 (53)
CALD background	6 (40)
Middle eastern	2
Asian	1
European	3
Language spoken at home	
English	9 (60)
English and other	3 (20)
NESB (interpreter required)	3 (20)
ASA score	
2	10 (66.7)
3	5 (33.3)
BMI, mean ± SD	33 ± 8.3
Employment status	
Full‐time	3 (20)
Part‐time	3 (20)
Retired	7 (46.7)
Home duties/carer	1 (6.7)
Disability pension	1 (6.7)
Living > 30 km from study site	4 (27)

**Healthcare professional participants (*n* = 15)**	***n* (%)**
Gender	
Male	9 (60)
Female	6 (40)
Length of interview, mean [range], time	0:41:34 [00:22:36–1:26:00]
Profession	
Physiotherapist	7 (46.7)
Orthopaedic surgeon	4 (26.7)
Anaesthetist	3 (20)
Surgical liaison nurse	1 (6.7)
Years of experience	
1 to 5	4 (26.7)
6 to 10	4 (26.7)
11 to 15	1 (6.7)
> 15	6 (40)

Abbreviations: ASA: American Society of Anaesthesiologists; BMI: body mass index; CALD: culturally and linguistically diverse; IQR: inter‐quartile range; NESB: non‐English speaking background; SD: standard deviation.

^a^ Only included participants who had received their joint arthroplasty surgery (post‐operative).

### 3.1 Themes

The following three major concepts were identified:

1. Understanding and beliefs around prehabilitation
2. Barriers and enablers influencing implementation of prehabilitation
3. Strategies for successful implementation of prehabilitation

Each concept encompassed several themes and sub‐themes. The sub‐themes within the first two concepts were mapped to the TDF (10/14 domains represented) and TFA (8/8 constructs represented). Sub‐themes of the third concept, ‘*strategies for success’,* encompassed potential solutions to the barriers identified in concept 2.

#### 3.1.1 Concept 1: Understanding and Beliefs Around Prehabilitation

This concept encompassed two primary themes: knowledge and attitude towards prehabilitation (four sub‐themes), and perceived goals and benefits of prehabilitation (three sub‐themes). These findings are presented in Table 2 and described in the narrative below. Additional quotes supporting each sub‐theme can be found in Supporting Information S1: eTable 5.

**TABLE 2:** Understanding and beliefs around prehabilitation, mapped to the TFA and TDF.

Item	Sub‐theme	No. of patients	No. of HCPs	Total no. of participants	TDF domains	TFA domains
**1. Knowledge of and attitude towards prehabilitation**						
1.1	There is varying awareness/knowledge of prehabilitation amongst both healthcare professionals and patients, which can impact acceptability (B/E)	15/15	15/15	30/30	Knowledge	Intervention coherence
1.2	Prehabilitation is generally viewed as a beneficial, low risk intervention (E)	14/15	12/15	26/30	Optimism	Affective attitude Perceived safety and risk
1.3	Difficulty exists in clarifying prehabilitation success and goals in this population (B) • Hard to define • Unclear measures of success • Lack of strong evidence	0/15	14/15	14/30	Knowledge	Perceived effectiveness Intervention coherence
1.4	For those offered prehabilitation, there was a sense of gratitude (E)	4/15	0/15	4/30	Emotions	Affective attitude
**2. Perceived goals and benefits of prehabilitation**						
2.1	Participating in prehabilitation improves post‐operative recovery (E)	10/15	13/15	23/30	Belief about consequences	Perceived effectiveness
2.2	There is a sense of control and empowerment through participating in prehabilitation (E)	9/15	4/15	13/30	Belief about capabilities	Self‐efficacy Opportunity gains
2.3	Belief that participating in prehabilitation has the potential to reduce resource use and clinician burden post‐operatively (E)	2/15	7/15	9/30	Belief about consequences	Opportunity gains Perceived effectiveness

*Note:* 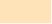 = Voiced by both professionals and patients; 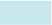 = Voiced by health professionals only; 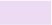 = Voiced by patients only.

Abbreviations: B/E, combined barrier and enabler; B: barrier; E: enabler; HCP, healthcare professional; TDF, Theoretical Domains Framework; TFA, Theoretical Framework of Acceptability.

##### 3.1.1.1 Knowledge of and Attitude Towards Prehabilitation

Understanding of prehabilitation varied among patients and HCPs. Some participants had little or no knowledge, while others understood that prehabilitation typically included physical optimisation, psychological preparedness, and education. Education about the benefits of prehabilitation and how to prepare for surgery was viewed as a minimum requirement for prehabilitation. **(1.1)** Patients with prior prehabilitation experience had greater awareness of what prehabilitation involved and appeared more likely to believe that it was effective. Conversely, those without any prehabilitation exposure lacked knowledge of the benefits, which seemed to influence their acceptability of the intervention.

I don’t know anything about [prehabilitation]. Someone would have had to do a bit of convincing to get me to do the prehab.

(P8—patient, male, 72, post‐TKA)

Among HCPs, physiotherapists appeared to have the greatest understanding and involvement with prehabilitation, whereas knowledge about prehabilitation was more variable among the surgeons and anaesthetists.

Both patient and HCP participants consistently believed that prehabilitation was a beneficial and low risk intervention for THA/TKA. Participants described feeling positively about the idea of prehabilitation and its anticipated benefits. **(1.2)**

I feel very positively about prehab. It gets you up and moving and it shows you that you can still do things.

(P3—patient, female, 68, post‐THA)

However, HCPs found defining prehabilitation success challenging due to limited evidence in this population, and difficulty differentiating the goals of prehabilitation from conservative management of osteoarthritis. **(1.3)**

I've had cases where they do prehab then they go “I don't want to have surgery anymore”… That really shouldn't be happening. Because then you've got this blurred line between prehab and conservative management, and what the goal is.

(P2—physiotherapist)

HCPs also highlighted uncertainty about what outcome measures indicate prehabilitation ‘success’. Patient‐reported outcomes were viewed as meaningful measures of success, while functional measures such as strength or complication rates, while considered useful, were felt to be less indicative of success.

I don't really know what [prehabilitation] success is. It's our best guess, we don't have an objective way of working out who will benefit and who won't. Patient‐reported outcomes is probably the most important, but it’s not a clean measurement, there’s other things that affect a PROM.

(P12—anaesthetist)

Patients who had been offered prehabilitation expressed feeling grateful that they had been given the opportunity to participate, as they felt supported in their preparation before the surgery. **(1.4)**

I was grateful when they offered me this prehab because I felt that people were interested in my own well‐being, they wanted to help me get through this surgery.

(P27—patient, male, 75, pre‐THA)

##### 3.1.1.2 Perceived Goals and Benefits of Prehabilitation

Most participants (> 75%) cited improved post‐operative recovery, including reduced length of hospital stay, enhanced rehabilitation participation, improved post‐surgical outcomes, and faster recovery/return to normal life, as the main goal of prehabilitation. **(2.1)**

Certainly [prehabilitation] is very important. Someone who is prepared mentally and physically will recover much better, faster.

(P28—patient, male, 74, pre‐TKA)

Patients who had completed a prehabilitation program appeared to have more realistic expectations of their recovery than those who did not. Many valued the continuity of care and ongoing engagement with the care team that prehabilitation offered.

It makes the transition a lot easier. They're so much more comfortable, knowing they’ve got to do these exercises, they’ve met the people in the team before. It just makes all the difference, particularly if they have lower health literacy.

(P9—physiotherapist)

Both patients and HCPs highlighted prehabilitation's role in building patient confidence and self‐empowerment, with patients reporting they felt like more active participants in their care. **(2.2)**

If people tell you what to do before your operation you feel like you've got a purpose.

(P13—patient, female, 66, pre‐TKA)

Both patient and HCP participants noted the potential for prehabilitation to reduce the need for post‐operative rehabilitation and, in some cases, to delay or avoid surgery. **(2.3)**

If I've got someone in the post‐operative group that has done prehab, I can get them started and get them going quicker and easier and then focus on the people that need it more…there’s less burden on me.

(P1—physiotherapist)

#### 3.1.2 Concept 2: Barriers and Enablers Influencing Implementation of Prehabilitation

This concept identified barriers and enablers influencing prehabilitation access, uptake and delivery at three levels of the care ecosystem: patient‐level (six sub‐themes), healthcare professional‐level (three sub‐themes) and system‐level (eight sub‐themes) levels (Table 3). Overall, eight barriers, two enablers, and seven mixed barriers and enablers were identified and summarised visually in Figure 1. Additional quotes supporting each sub‐theme can be found in Supporting Information S1: eTable 6.

**TABLE 3:** Barriers and enablers influencing implementation of prehabilitation in the joint arthroplasty population, mapped to the domains of the TDF and TFA.

Item	Sub‐theme	No. of patients	No. of HCPs	Total no. of participants	TDF domains	TFA domains
**3. Patient‐related implementation factors**						
3.1	Physical capability influences ability to participate in prehabilitation (B/E)	9/15	6/15	15/30	Skills Beliefs about capabilities	Self‐efficacy
3.2	Previous experience with exercise or surgery influences uptake (B/E)	4/15	2/15	6/30	Skills Beliefs about capabilities	Self‐efficacy
3.3	Patient belief system/mindset impacts uptake (B/E)	15/15	13/15	28/30	Belief about consequences Optimism	Affective attitude Self‐efficacy
3.4	A individuals' situation/background can impact acceptability and uptake (B/E): • CALD background • Travel • Financial • Employment/availability • Social roles	11/15	11/15	22/30	Environment, context and resources Social/professional role and identity	Opportunity costs Burden
3.5	Social environment can impact uptake (E) • Peer support/role models • Family and friends	7/15	2/15	9/30	Social influences	Opportunity gains
3.6	Implicit trust in the care teams recommendations promotes uptake (E)	12/15	0/15	12/30	Social influences	*Trust
**4. Healthcare professional‐related implementation factors**						
4.1	Healthcare professionals' confidence in discussing/delivering prehabilitation is linked with knowledge and experience (B/E)	0/15	13/15	13/30	Skills Belief about capabilities Knowledge	Self‐efficacy Intervention coherence
4.2	Lack of clarity exists regarding professional roles and involvement in prehabilitation pathway, and how the care team works together (B)	0/15	11/15	11/30	Social and professional role and identity	*Trust
4.3	Time is a barrier to discussing/delivering prehabilitation (B)	0/15	9/15	9/30	Environment, context and resources	Opportunity costs
**5. System‐related implementation factors**						
5.1	Timing of prehabilitation relative to surgery is challenging due to unpredictable surgery dates and prolonged waitlists (B)	9/15	14/15	23/30	Environment, context and resources Emotions	Affective attitude
5.2	The current care pathway lacks communication and monitoring between patient and health service (B)	7/15	9/15	16/30	Environment, context and resources Emotions	Affective attitude
5.3	Access to information and resources in the pre‐operative time is desired and valued when received but can be lacking (B/E)	14/15	11/15	25/30	Environment, context and resources Emotions	—
5.4	Prehabilitation referral processes are unclear and programs are thought to be difficult to access (B)	0/15	8/15	8/30	Environment, context and resources Memory, attention, processes	—
5.5	Tensions exist between equitable access and ethical autonomy in prehabilitation (B)	6/15	6/15	12/30	Environmental context and resources	Ethicality
5.6	There is uncertainty about value and patient selection in the context of scarce resources (B)	0/15	9/15	9/30	Beliefs about consequences Memory, attention and processes	Burden Opportunity costs Ethicality
5.7	Pre‐operative care should be tailored to individual needs, however it is not currently needs‐based (B/E)	8/15	14/15	22/30	Environmental content and resources	—
5.8	Access to multi‐disciplinary team members is desired but difficult (B)	3/15	5/15	8/30	Environment, context and resources	—

*Note:* 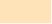 = Voiced by patients only; 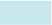 = Voiced by health professionals only; 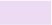 = Voiced by both professionals and patients. *: identified domain that did not fit within the original TFA framework.

Abbreviations: B/E: combined barrier and enabler; B: barrier; E: enabler; HCP: healthcare professional; TDF: Theoretical Domains Framework; TFA: Theoretical Framework of Acceptability; CALD: culturally and linguistically diverse.

**FIGURE 1:**
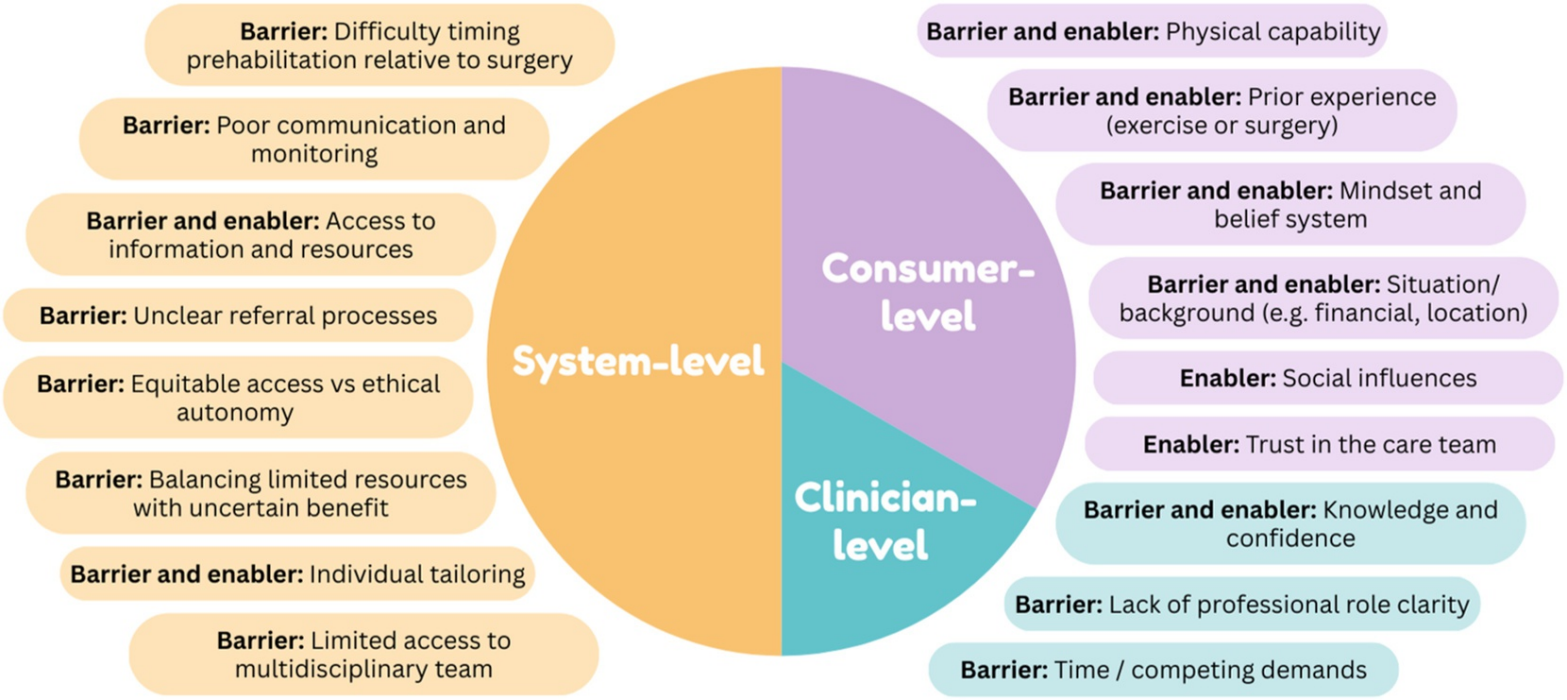
Barriers and enablers identified at different levels of the care ecosystem impact prehabilitation implementation.

##### 3.1.2.1 Patient‐Related Implementation Factors

Six factors influencing an individuals' likelihood of participating in prehabilitation were identified at the patient level. Most were reported by both the patient and HCP groups, and represented both barriers and enablers to prehabilitation uptake and delivery.

An individual's physical capability and confidence to participate in prehabilitation was a common factor impacting uptake. Pain, fear of pain, and physical disability due to joint disease commonly discouraged participation. **(3.1)**

I was in a lot of pain, I don’t know if I would’ve actually been able to do any prehab if it was offered.

(P25—patient, female, 79, post‐TKA)

Prior positive experiences with exercise or surgery were reported as enabling factors, through promoting skill development and self‐confidence. **(3.2)** In contrast, limited or poor experiences tended to prevent engagement with prehabilitation, highlighting how experiential gains in self‐efficacy shape acceptability of the intervention.

Prior experience is a classic barrier, ‘I've tried Physio, didn't work’.

(P19—surgeon)

Intrinsic mindsets and belief systems strongly influenced prehabilitation acceptance, functioning as either barriers or enablers. Participants with high motivation, internal drive, or positive beliefs about exercise were more likely to seek information and opportunities to engage. **(3.3)**

I'll do anything to get myself right. I've got a goal. That comes from your own mental attitude.

(P3—patient, female, 68, post‐THA)

In contrast, patients who believed that surgery was the only ‘fix’ and exercise could be ‘damaging’ were less likely to participate in prehabilitation.

People's belief system about well, I have arthritis, therefore I shouldn't exercise is also a limiting factor.

(P4—physiotherapist)

Patient's circumstances and environment also shaped prehabilitation engagement. Key barriers included financial cost (related to travel or private service access), geographic location limiting in‐person participation, and time constraints (often related to work). These factors underscored the personal burden associated with participation for some. **(3.4)**

Because of the distance between where I live and the hospital, and because I am still working, it was too hard to come in to do the prehab.

(P7—patient, male, 56, post‐THA)

Several HCPs reported that culturally and linguistically diverse individuals were disadvantaged, with a lack of translated resources and interpreting services resulting in poor engagement with prehabilitation.

But for the patient who is culturally, linguistically diverse and can't come into clinic, there's then a significant limitation on what we can offer them. Because we don't have translated resources, we don't have an easily accessible portal.

(P23—physiotherapist)

Social and familial roles also influenced uptake. One participant noted that prehabilitation would need to fit around caregiving duties, while another was motivated to participate so she could recover quickly and resume caring for her children.

Several social influences enabled engagement. Patients who had completed prehabilitation highlighted the motivating effect of peer support and shared experiences. **(3.5)**

When you can get that information from someone that's just like you, that’s gone through the same things, that is really powerful.

(P5—patient, female, 67, post‐THA)

Participants reported that family and friends were typically supportive of prehabilitation, though patients emphasised that participation was ultimately their decision.

[My family] were all for [prehabilitation]. They encouraged it. Although I would have done it regardless.

(P5—patient, female, 67, post‐THA)

Patients expressed implicit trust in the recommendations of the care team, particularly the orthopaedic surgeon. When prehabilitation was recommended as part of the surgical pathway, patients were more likely to participate. This underscores the importance of trust in HCPs recommending and delivering the intervention. **(3.6)**

Once you've been told by a surgeon, then it's like the surgeon told me I have to do it. I trust them. But if it's somebody not that high up on the hierarchy, I might not be as likely to listen.

(P24—patient, female, 52, post‐THA)

##### 3.1.2.2 Healthcare Professional‐Related Implementation Factors

Voiced exclusively by HCP participants, three factors influencing referral and delivery of prehabilitation prior to THA/TKA were identified.

Knowledge about prehabilitation and confidence to discuss it facilitated referral to prehabilitation. **(4.1)** In contrast, lack of knowledge about prehabilitation was identified as a barrier to discussing it with patients.

I feel like a non‐expert in prehab so I don’t really feel equipped to have that conversation.

(P1‐ anaesthetist)

HCPs noted that the lack of clarity about roles and responsibilities in relation to prehabilitation often led to inconsistent referring practices and care. To promote interprofessional trust and coordinated care, they emphasised the need for improved role clarity and delineation. **(4.2)**

Anaesthetists can definitely have a role in promoting prehab. But do we feel empowered to suggest prehab?… Is it ok for me to talk to them about prehabiltiation? Or does the surgeon just want them operated on?

(P20—anaesthetist)

HCPs reported working in discipline‐specific silos with little knowledge of the involvement of other team members in prehabilitation was.

I don't know whether [the surgeons] talk about prehab at all. I have very little contact with [the multi‐disciplinary team].

(P2—physiotherapist)

Limited time in consultations and competing demands were raised by surgeons and anaesthetists as barriers to discussing or referring to prehabilitation. **(4.3)**

We have 10‐ or 15‐minute slots. There's no way you can fit it all in.

(P22—surgeon)

Time constraints due to inefficient processes were also raised by physiotherapists regarding assessment for, and delivery of, prehabilitation, impacting work satisfaction.

##### 3.1.2.3 System‐Related Implementation Factors

Participants highlighted several systemic factors affecting their capacity to engage in prehabilitation. Eight system‐level sub‐themes were identified. These mostly mapped to the TDF domain ‘*environmental context and resources’*, underscoring the impact of infrastructure on behaviour.

A key system‐level barrier was the timing of prehabilitation relative to surgery. Both HCPs and patients commented that unpredictable surgery dates and unclear booking processes impeded their ability to provide information and deliver the intervention. **(5.1)**

The reason I don't get involved as much is cause of the time frame. There's no point writing out all these referrals [for prehabilitation] if they're just gonna go in a pile and you don't know exactly when they're gonna get their surgery.

(P19—surgeon)

Acknowledging that surgery dates are often provided only 3–4 weeks before the surgery, participants felt that starting prehabilitation after this point would not allow enough time for it to be effective. Conversely, they believed that completing prehabilitation well before surgery could negatively affect motivation and attitudes towards prehabilitation.

After 3 months of doing the prehab, to be told I was probably looking at another 6–12 months until surgery, I just fell into a bit of a hole. I felt like I gave up.

(P7—patient, male, 56, post‐THA)

Both participant groups described communication between the health service and patients as insufficient. Patients reported that poor pre‐operative communication contributed to feelings of isolation and negatively affected their mental and physical health. **(5.2)**

There was just no communication, it felt really lonely in that time before the operation.

(P16—patient, male, 71, post‐TKA)

Similarly, HCPs reported a similar disconnect during the pre‐operative period, expressing an inability to monitor a patient's condition and intervene accordingly.

I think the hard thing for us as surgeons is that once they're on the waiting list, we don't tend to see them until the surgery, and this is a problem. We're not able to monitor their condition.

(P17—surgeon)

While some patients felt adequately informed or preferred limited information, others reported unmet education needs, causing apprehension and a fear of the unknown. **(5.3)**

I would’ve liked to know more about what to expect to feel like afterwards. That would have helped me mentally… It feels like you’re going in blindly.

(P24—patient, female, 52, post‐THA)

HCPs noted, and several patients agreed, that information is often poorly retained during appointments, highlighting the need for educational material to take home.

But we just don't have the physical information to give to patients.

(P21—surgical liaison nurse)

Low health‐literacy, language barriers, and limited CALD‐accessible resources were reported to hinder engagement with pre‐operative education.

Exercise equipment and hydrotherapy facilities were valued highly by participants, whilst acknowledging that access to these resources varied between healthcare services.

We've got access to really good equipment like a gym space and access to the hydro pool. But I know other healthcare networks might not have that luxury of having the facilities available.

(P9—physiotherapist)

HCPs reported that the referral process for prehabilitation programs, both at the study site and more broadly in the community, was unclear and a barrier to referring. **(5.4)**

But if I knew of a specific referral pathway then I would probably refer them. But if there's no referral pathway, I probably wouldn't because I don't see any way that I can actually send them to prehab.

(P10—surgeon)

HCPs reported that limited availability and long wait times for community prehabilitation, especially for patients living far from the study site, hindered referrals and was a common source of frustration.

Both patient and HCP participants voiced dissatisfaction that prehabilitation is not offered to everyone, with general agreement that everyone should be offered prehabilitation as standard care. **(5.5)**

I feel like everyone should have the equal opportunity to complete prehab. But that's certainly not how it is currently.

(P9—physiotherapist)

However, there were some ethical concerns raised, with some HCPs questioning whether prehabilitation should be a mandatory requirement before surgery.

For me a big ethical question is do patients have the capacity to refuse prehabilitation? Do they really understand the benefits? Which then puts us in an ethical quandary, maybe we need to say it's something you have to do and you can't get the operation without it.

(P20—anaesthetist)

Some HCPs described feeling concerned about how scarce healthcare resources should be allocated, with uncertainty about who benefits from prehabilitation and whether offering it universally could negatively impact other health service areas. **(5.6)**

From a health economics side, are you taking away from something else? Can we use that money elsewhere that's better?

(P18—surgeon)

Participants emphasised the importance of targeting prehabilitation to those most likely to benefit, such as those who are overweight, less active, have lower health literacy, or are more anxious. HCPs wanted an objective screening tool to advise who should be referred to the service, postulating that resources are not currently being well utilised. **(5.7)**

My gut feel is that we're spending the majority of our time on the people who don't need us.

(P23—physiotherapist)

Both patients and HCPs wanted prehabilitation to be tailored to individual needs, risk profiles, and co‐morbidities.

We don’t screen for risk and provide a patient‐specific prehab service. Prehab should adapt to the person, not the other way around

(P1—physiotherapist)

Multi‐disciplinary team (MDT) care was identified as integral to delivering needs‐based prehabilitation. Access to physiotherapists was considered essential for exercise prescription and supervision. **(5.8)** However, limited access to other MDT members, particularly psychology and dietetics, was highlighted as a major barrier to achieving multi‐faceted prehabilitation care.

From my experience in the pre‐admission setting, there's been no access to get these patients to psych, or dietitians for weight loss, to prepare them for surgery.

(P11—physiotherapist)

#### 3.1.3 Concept 3: Strategies for Successful Implementation of Prehabilitation

The third concept focused on implementation strategies to improve how prehabilitation would be delivered. Patients and HCPs expressed concordant views on what ideal prehabilitation would include, with core suggestions grouped into sub‐themes addressing ‘what’, ‘when’, ‘who’, and ‘how’. Table 4 summarises these sub‐themes and the corresponding implementation strategies suggested by participants, and links them to identified barriers and enablers in Concept 2. Overall, ten sub‐themes for improving prehabilitation prior to THA and TKA were identified (Figure 2). Additional quotes supporting each sub‐theme can be found in Supporting Information S1: eTable 7.

**TABLE 4:** Sub‐themes relating to strategies for successful orthopaedic prehabilitation, barriers and/or enablers related to the sub‐theme, and proposed implementation strategies.

Sub‐themes	Related barrier/enabler	Proposed implementation strategies
**What should prehabilitation involve?**		
Empower with education	*Access to information and resources in the pre‐operative time is desired and valued when received but can be lacking* ***(5.3)*** *Patient belief system/mindset impacts uptake* ***(3.3)***	Develop comprehensive, individualised education across the waitlist journey incorporating in‐person, online and paper‐based models Develop culturally and linguistically diverse appropriate resources (available in multiple languages) Provide education topics to facilitate recovery provided across a series of prehabilitation program sessions (e.g., purpose of prehabilitation, types of anaesthetic, pain expectations and management, what to expect post‐operatively)
Peer support	*Social environment can impact uptake* ***(3.5)***	Group‐based prehabilitation program with peer support or access to support groups for patients who desire this (consider pre‐recorded video stories and/or face‐to‐face/telehealth models) Video vignettes of patient journey including benefits of prehabilitation provided online—share experiences of common symptoms and setbacks Monitored online chat for sharing experiences
Clearly defined goals	*Difficulty exists in clarifying prehabilitation success and goals in this population* ***(1.3)***	Differentiate goals of conservative management of osteoarthritis versus goals of prehabilitation Agreement on outcome measures to measure effect of prehabilitation at the individual and population level
**When should prehabilitation be delivered?**		
Throughout the waitlist journey	*Timing of prehabilitation relative to surgery is challenging due to unpredictable surgery dates and prolonged waitlist* ***(5.1)***	“Slow stream” and “fast stream” prehabilitation—begin discussions and provide access to general advice early, followed by “top‐up” prehabilitation through a formal supervised program closer to surgery Regular reinforcement of previously provided education Develop online resources patients can refer back to throughout the waitlist journey
Responsive and adaptive	*The current care pathway lacks communication and monitoring between patient and health service* ***(5.2)***	Implement an online system to remotely monitor patients throughout the waitlist period and improve communication between the patient and the health service (e.g., patient accessible portal with resources, remotely collected outcome measures and chat function) Use patient‐reported outcome measures to monitor mental and physical condition over time and adapt prehabilitation content to changing needs
**Who should be targeted and/or involved?**		
Tailored to the individual	*There is uncertainty about value and patient selection in the context of scarce resources* ***(5.6)*** *Pre‐operative care should be tailored to individual needs, however it is not currently needs‐based* ***(5.7)***	Implement formal screening to identify modifiable risk factors (e.g., frailty, body mass index, mental health status, exercise capacity) and individual education/support needs to guide care needs Criteria‐based referrals with clear guidelines Goal‐based prehabilitation targeting patient‐centred goals
Multi‐disciplinary approach	*Access to multi‐disciplinary team members is desired but difficult* ***(5.8)*** *Prehabilitation referral processes are unclear and programs are thought to be difficult to access* ***(5.4)***	Provide access/links to diet and mental health professionals for support Engage key multi‐disciplinary team members for inclusion in multi‐modal prehabilitation program (e.g., Physiotherapist, dietician, psychologist, anaesthetist, orthopaedic surgeon) Routine access to allied health pre‐operative assessment and education Clarify referral pathways to multi‐disciplinary team members within and external to healthcare service Buy‐in from medical/surgical teams to facilitate their encouragement of participation in prehabilitation
Coordinated care team	*Healthcare professionals' confidence in discussing/delivering prehabilitation is linked with knowledge and experience* ***(4.1)*** *Lack of clarity exists regarding professional roles and involvement in prehabilitation pathway, and how the care team works together* ***(4.2)***	Include a care coordinator role within the program—dedicated role that provides oversight of the entire prehabilitation program Education to the wider multi‐disciplinary team about individual roles in the care pathway and development of a common language (including general practitioners, allied health, and medical staff) Provide education sessions to staff around prehabilitation and services offered Motivational interviewing training to effectively explore patient‐related barriers and enablers to prehabilitation participation
**How should prehabilitation be delivered?**		
Multi‐modal delivery	*A individuals' situation/background can impact acceptability and uptake* ***(3.4)***	Offer prehabilitation program at alternative venues (e.g., partner with community services) Offer home‐based or telehealth prehabilitation options where appropriate
Operational efficiency	*Time is a barrier to discussing/delivering prehabilitation* ***(4.3)***	Utilise digital platforms to reduce duplicity of information (e.g., patient‐accessible portal to deliver patient education) Use of checklists in consultations Automated texts/reminders

**FIGURE 2:**
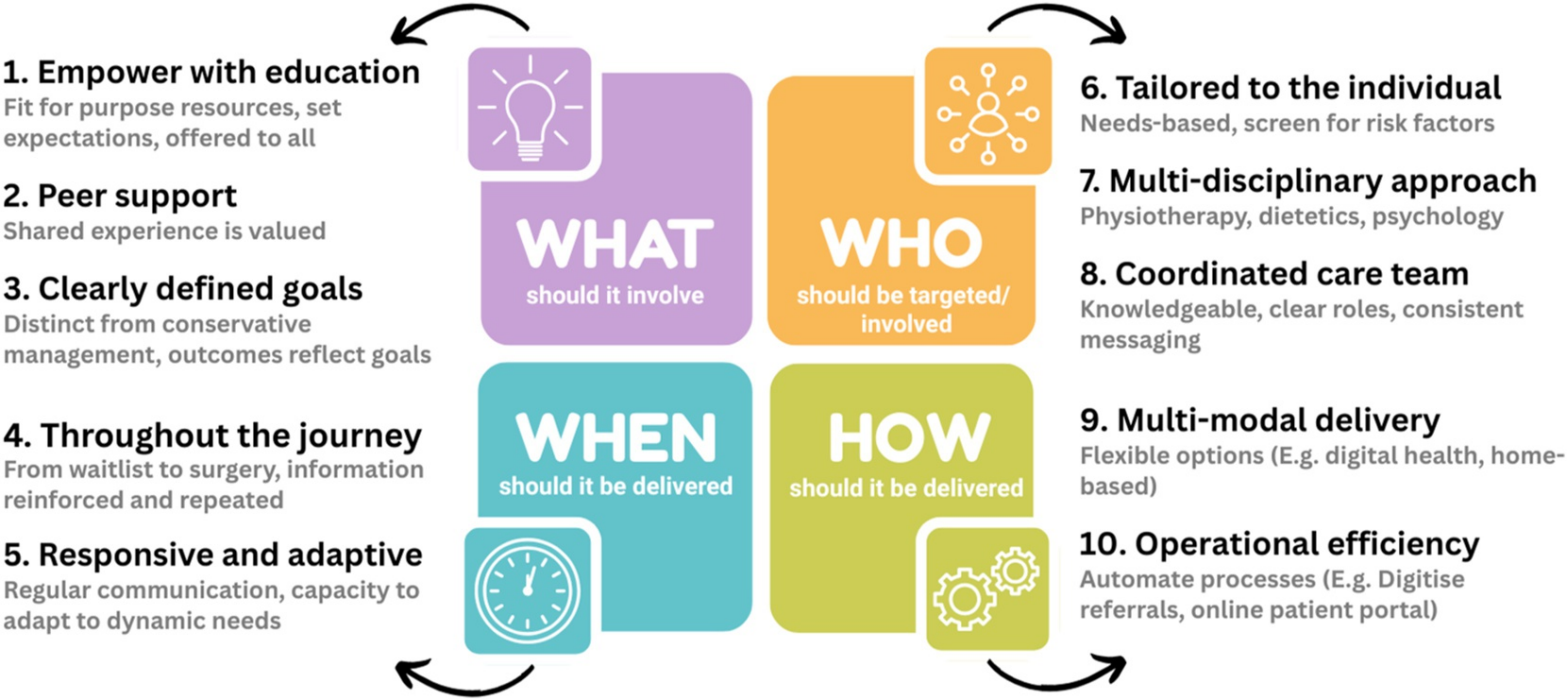
Ten recommendations to support future implementation of prehabilitation prior to total hip and knee arthroplasty.

##### 3.1.3.1 What Should be Involved?

Education was identified as a core component of prehabilitation, with patients emphasising the need for clear and credible information regarding post‐operative expectations, nutrition, mental health, and prehabilitation benefits. They wanted the information to be tailored to individual needs, accommodating low health literacy and language barriers, and delivered in multiple formats (e.g., print, online).

Patients valued interacting with peers that were in a similar situation and viewed peer support as an important component of prehabilitation.

I would suggest having a session where a number of people get together and explain what the benefits are and what your issues are, and you can learn from each other.

(P8—patient, male, 72, post‐TKA)

HCPs highlighted the importance of clearly defining prehabilitation, with goals and outcomes distinct from those of conservative management.

We need to differentiate and have really clear goals and objectives of prehab and conservative management.

(P23—physiotherapist)

##### 3.1.3.2 When Should it be Delivered?

Participants highlighted that elective joint arthroplasty is uniquely characterised by prolonged waiting periods, and advocated for prehabilitation to span the care continuum from waitlisting to surgery, with evolving intensity and focus.

Probably we need to do a 2‐speed thing, so you get put on [the waitlist], get given the one pager with ‘this is what you can do for yourself’. Maybe contact them again three monthly. And then you kick it up a gear closer to surgery, that’s where you get them to come in and do a supervised program.

(P18—surgeon)

Certainly I would want information over a period of time. So not just that once, not just right when you're gonna go in for the op[eration].

(P16—patient, male, 71, post‐TKA)

Both groups supported a responsive model that monitors progress remotely, and adapts to changing needs during the waitlist period.

I think we need to be monitoring [patients] whilst they’re on the waitlist, and I think there is an enormous opportunity here for the utilisation of [patient reported outcome measures] to monitor significant patient deterioration, and then respond accordingly.

(P23—physiotherapist)

Clear and timely communication regarding surgery scheduling was considered essential to optimise engagement.

I think there should be more communication between the patients and system, just to check in and keep you updated. Because it's a scary time for all of the patients.

(P16—patient, male, 71, post‐TKA)

##### 3.1.3.3 Who Should be Targeted/Involved?

Participants advocated for risk‐stratified, individualised prehabilitation supported by clear referral pathways to optimise resource allocation and address specific patient needs.

It’s about providing the best service, tailored to what they need.

(P1—physiotherapist)

Whilst physiotherapists were considered central to delivery, prehabilitation was considered inherently multidisciplinary, requiring access to professionals such as psychologists and dieticians to address broader well‐being.

There should be a team including dietetics, psychology, maybe pain specialists, physio…there need to a be a dedicated multidisciplinary clinic that we can access.

(P18—surgeon)

Consistent messaging from all HCPs involved in joint replacement care was deemed essential. Appointing a care coordinator to oversee the program was suggested to help achieve this.

It would be good to have a person that kind of coordinates everything and has access to the surgical team. They need to be an expert across multiple fields, I'd say.

(P2—physiotherapist)

##### 3.1.3.4 How Should it be Delivered?

Perspectives on delivery methods differed. HCPs favoured digital options (e.g., online resources and telehealth) to improve access and efficiency, whereas patients preferred in‐person sessions for personalisation and peer support, with options to access community‐based services closer to home. While some patients lacked confidence and skills in using digital modalities, others supported a hybrid model (face‐to‐face and online delivery) to provide flexibility for those working or living remotely.

I’m a big believer in in‐person…But if you could give me a program and every week I touch base on telehealth and say ‘yes, I've done my program this week’ instead of having to come all the way here. That's an option.

(P7—patient, male, 56, post‐THA)

Streamlining services through automated referrals or digitising resources was suggested to reduce HCP burden.

The more laborious the referral is that's going to be a barrier…It should just be automated.

(P10—anaesthetist)

## 4 Discussion

This study indicates that prehabilitation prior to THA and TKA is generally well‐accepted by both patients and HCPs. Barriers and enablers were identified at the patient, HCP and system levels of care, with most barriers existing at the system level. Findings highlight the complexity of implementing prehabilitation within healthcare systems, and the need for coordinated, multi‐level implementation strategies.

Applying the TFA illuminated the importance of knowledge in shaping acceptability and the need for enhanced education across stakeholder groups. Consistent with prior research in orthopaedic and non‐orthopaedic populations (Khalid et al. 2024; Powell et al. 2023; Smyth et al. 2024), participants perceived prehabilitation as safe and beneficial for improving postoperative outcomes. Trust emerged as a salient construct not explicitly captured within the TFA. Trust in the care team (for patients) and trust within and between professional groups (for HCPs) influenced perceptions of prehabilitation acceptability. While the TFA addresses self‐efficacy, the findings suggest that acceptability also requires the participant to have confidence in those delivering the intervention. Others have similarly demonstrated the importance of trust in intervention acceptability (Corbaz‐Kurth et al. 2025; Dyer et al. 2016; Haughton et al. 2025).

Factors influencing the uptake and delivery of prehabilitation mapped to the TDF, and aligned with previous qualitative research. At the patient level, intrinsic motivation, beliefs about consequences, previous experiences, and physical capability have similarly been identified as influencing engagement with prehabilitation programs prior to orthopaedic surgery (Halsen et al. 2023; Schöne et al. 2025). Barriers including travel distance, time constraints, and cultural and linguistic considerations echo existing literature (Fuchs et al. 2024; Schöne et al. 2025; van der Velde et al. 2023), emphasising the need for adaptable program models. In our study, digital delivery modes were suggested to improve access; however, concerns regarding reduced social support, limited supervision, and technology literacy were raised. Similar findings have been reported in studies of digitally‐delivered prehabilitation for THA and TKA, where online formats were acceptable but constrained by limited personalisation and computer literacy (Anderson et al. 2025; Mohammadi et al. 2021). Hybrid models that combine digital delivery with opportunities for real‐time interaction, for example video‐teleconferencing or mobile applications with real‐time support from exercise professionals, may address these limitations by retaining the flexibility and accessibility of remote participation while providing opportunities for individualised guidance, feedback, troubleshooting and social support. These features may enhance patients' confidence in performing exercises, facilitate adaptation of prehabilitation to individual needs, and strengthen engagement over time.

At the HCP, limited knowledge and confidence in discussing prehabilitation and uncertainty regarding professional roles within the care pathway impeded implementation, consistent with findings in non‐orthopaedic populations (Chapman 2021). These findings underscore the need for HCP training and upskilling, and the value of care coordination frameworks. Participants called for a dedicated prehabilitation coordinator to streamline care and enhance communication, as well as multidisciplinary team collaboration. This reflects recommendations from previous prehabilitation implementation studies suggesting that a multidisciplinary approach with clear communication and cooperation between professional groups is integral to providing cohesive and holistic care (Fuchs et al. 2024; Rossen et al. 2025; Sontag et al. 2024).

Limited resources and workforce capacity have previously been identified as major impediments to prehabilitation implementation (Chapman 2021; Sontag et al. 2024), consistent with the present study. At the system‐level, barriers included limited access to information, resources, and multi‐disciplinary team members. Participants advocated for improved pre‐operative educational resources offered to all, addressing the purpose of prehabilitation, hospital expectations, pain management, and recovery. These priorities are consistent with international consensus recommendations on pre‐operative education and prehabilitation for TKA (Anderson et al. 2021). Pre‐operative education is also an established component of Enhanced Recovery After Surgery (ERAS) pathways for total hip and knee arthroplasty, where it is recommended alongside other elements of perioperative optimisation (Wainwright et al. 2020). Similarly, in the Macmillan guidelines of prehabilitation for people with cancer, ‘universal prehabilitation’ has been defined as the provision of expert advice on exercise, nutrition and psychological support along with behaviour change advice to all individuals, and is the foundation of prehabilitation (Macmillan Cancer Support 2025). Pre‐operative education has been evaluated as a standalone intervention in patients undergoing total hip and knee arthroplasty, and found to decrease post‐operative pain, reduce pre‐operative anxiety, and reduce length of stay (Longo et al. 2023). Co‐design of educational materials and delivery formats with stakeholders should be prioritised to ensure relevance and accessibility (Brand et al. 2025).

Participants underscored the importance of personalised, risk‐stratified prehabilitation, echoing previous findings (Rossen et al. 2025; Schöne et al. 2025). Existing literature supports risk screening and tailored interventions prior to THA and TKA to deliver needs‐based care (Karimijashni et al. 2024; Vervullens et al. 2023); however, the absence of validated screening tools, limited clarity regarding which patients benefit most, and uncertainty regarding optimal program components, remain key research gaps.

The timing of prehabilitation within the context of prolonged public‐system waitlists for THA and TKA (Ackerman 2022) was a novel barrier. Extended wait times may lead to prehabilitation being completed too early, potentially leading to psychological burden and reduced perceived value. Participants proposed a longitudinal, responsive model extending from surgical consent to the operation, challenging conventional 2–8 week models traditionally evaluated in THA and TKA trials (Burgess et al. 2025). This aligns with previous qualitative evidence supporting regular follow‐up and coping strategies to facilitate the concept of ‘waiting well’ (Chan et al. 2025). Expanding conceptualisation of prehabilitation links to questions raised in the present study regarding its definition, measures of success and scope, including whether it is distinct from or contiguous with conservative osteoarthritis management. These findings reflect heterogeneity in definitions and outcomes reported in prehabilitation trials for THA and TKA (Burgess et al. 2025). A reframing of exercise‐based prehabilitation towards ‘pre‐evaluation’ to inform surgical decision‐making has been proposed (Bandholm et al. 2025). Future work remains to clarify whether prehabilitation should prioritise postoperative outcomes, quality of life during waiting periods, or optimisation and potential diversion from surgery where appropriate.

This study has several strengths. Extending the existing qualitative evidence generated within specific prehabilitation trials, this study explored prehabilitation from a broader perspective, generating insights relevant to the development and optimisation of future models of care. Inclusion of patients and HCPs with diverse backgrounds, including culturally and linguistically diverse participants, enabled triangulation of perspectives. The use of two established implementation frameworks enhanced methodological rigour.

Limitations include predominantly urban recruitment, which may limit transferability to regional settings, and being conducted within a single quaternary public hospital, which may not reflect other settings. Finally, patients may have held favourable views towards prehabilitation, as recruitment of individuals who declined prehabilitation was unsuccessful.

## 5 Conclusion

This study provides detailed insights into patient and HCP perceptions of prehabilitation prior to THA and TKA, including factors shaping acceptability and implementation. Findings highlight the need for improved education, care coordination, and personalised intervention design. These insights offer practical direction for a stakeholder‐informed prehabilitation model and future implementation strategies.

## Author Contributions

All authors were involved in conceptualising the study. N.B and M.G developed the interview guide. N.B completed all participant recruitment and conducted all interviews. N.B, E.W and M.G analysed the data. S.B, L.D and L.E provided project administration and supervision. N.B drafted the original manuscript. All authors contributed to and approved the final manuscript.

## Funding

The authors have nothing to report.

## Ethics Statement

Ethical approval for this research was granted by the Human Research Ethics Committees at Austin Health in October 2024 (#109358). All participants provided informed consent to participate and for their anonymised data to be used in publication of this research.

## Conflicts of Interest

The authors declare no conflicts of interest.

## Supporting information


Supporting Information S1

## Data Availability

The data that support the findings of this study are available on request from the corresponding author. The data are not publicly available due to privacy or ethical restrictions.

## References

[msc70266-bib-0001] Abujaber, S. , I. Altubasi , M. Hamdan , and R. Al‐Zaben . 2023. “Impact of End‐Stage Knee Osteoarthritis on Perceived Physical Function and Quality of Life: A Descriptive Study From Jordan.” PLoS One 18, no. 6: e0286962. 10.1371/journal.pone.0286962. PMC10256207 37294813

[msc70266-bib-0002] Ackerman, I. N. 2022. “Waiting for Orthopedic Surgery—Are We Underestimating the Impacts on People With Advanced Osteoarthritis?” Osteoarthritis and Cartilage 30, no. 12: 1545–1546. 10.1016/j.joca.2022.08.012. PMC9458548 36063989

[msc70266-bib-0003] Adebero, T. , H. Omana , L. Somerville , B. Lanting , and S. W. Hunter . 2024. “Effectiveness of Prehabilitation on Outcomes Following Total Knee and Hip Arthroplasty for Osteoarthritis: A Systematic Review and Meta‐Analysis of Randomized Controlled Trials.” Disability & Rehabilitation 46, no. 24: 5771–5790. 10.1080/09638288.2024.2313128. 38349251

[msc70266-bib-0004] Anderson, A. M. , C. Comer , T. O. Smith , et al. 2021. “Consensus on Pre‐Operative Total Knee Replacement Education and Prehabilitation Recommendations: A UK‐Based Modified Delphi Study.” BMC Musculoskeletal Disorders 22, no. 1: 352. 10.1186/s12891-021-04160-5. PMC8044503 33853564

[msc70266-bib-0005] Anderson, A. M. , A. C. Redmond , J. Joseph , and G. A. McHugh . 2025. “‘I'mMa pPper and pPncil pPrson’: A qQalitative dDscriptive study of pPtential bBrriers and fFcilitators to eEgagement With pPe‐operative tTtal kKee rRplacement eEucation and pPehabilitation dDgital iIterventions.” BMC Musculoskeletal Disorders 26, no. 1: 652. 10.1186/s12891-025-08673-1. PMC12228215 40616014

[msc70266-bib-0006] Bandholm, T. , R. S. Husted , A. Troelsen , and K. Thorborg . 2025. “Changing the Narrative for Exercise‐Based Prehabilitation: Evidence‐Informed and Shared Decision Making When Discussing the Need for a Total Knee Arthroplasty With Patients.” Osteoarthritis and Cartilage Open 7, no. 2: 100601. 10.1016/j.ocarto.2025.100601. PMC11960630 40170680

[msc70266-bib-0007] Brand, G. , C. Sheers , A. Hansen , et al. 2025. “How to… Co‐Design Education With Healthcare Consumers.” Clinical Teacher 22, no. 2: e70039. 10.1111/tct.70039. PMC11826370 39950236

[msc70266-bib-0008] Braun, V. , and V. Clarke . 2006. “Using Thematic Analysis in Psychology.” Qualitative Research in Psychology 3, no. 2: 77–101. 10.1191/1478088706qp063oa.

[msc70266-bib-0009] Burgess, N. , S. N. Voelker , B. Phillips , et al. 2025. “Outcome Measures in Prehabilitation Interventions for Total Hip and Knee Arthroplasty: A Scoping Review.” Clinical Rehabilitation 40, no. 1: 44–68. 10.1177/02692155251378374. PMC12722570 40971560

[msc70266-bib-0010] Cane, J. , D. O’Connor , and S. Michie . 2012. “Validation of the Theoretical Domains Framework for Use in Behaviour Change and Implementation Research.” Implementation Science 7, no. 1: 37. 10.1186/1748-5908-7-37. PMC3483008 22530986

[msc70266-bib-0011] Chan, J. , S. Poon , A. Lawrence‐Jones , et al. 2025. “Patient Experiences of Waiting for Orthopaedic Care and Priorities for ‘Waiting Well’: A Qualitative Study in a London NHS Trust.” Archives of Public Health 83, no. 1: 95. 10.1186/s13690-025-01578-4. PMC11974020 40197335

[msc70266-bib-0012] Chapman, O. T. R. 2021. “Barriers and Facilitators for Healthcare Professionals to Implementating a Prehabilitation Programme: Review of the Literature.” Journal of Cancer Rehabilitation 4: 86–90. 10.48252/JCR32.

[msc70266-bib-0013] Clode, N. J. , M. A. Perry , and L. Wulff . 2018. “Does Physiotherapy Prehabilitation Improve Pre‐Surgical Outcomes and Influence Patient Expectations Prior to Knee and Hip Joint Arthroplasty?” International Journal of Orthopaedic and Trauma Nursing 30: 14–19. 10.1016/j.ijotn.2018.05.004. 29954717

[msc70266-bib-0014] Corbaz‐Kurth, S. , R. Weissbrodt , T. M. Juvet , et al. 2025. “How Does Trust Emerge in Interprofessional Collaboration? A Qualitative Study of the Significance, Importance, and Dynamics of Trust in Healthcare Teams and Networks.” Journal of Interprofessional Care 40, no. 5: 1–10. 10.1080/13561820.2025.2495013. 40370201

[msc70266-bib-0015] Daigle, M. E. , A. M. Weinstein , J. N. Katz , and E. Losina . 2012. “The Cost‐Effectiveness of Total Joint Arthroplasty: A Systematic Review of Published Literature.” Best Practice & Research Clinical Rheumatology 26, no. 5: 649–658. 10.1016/j.berh.2012.07.013. PMC3879923 23218429

[msc70266-bib-0016] Dyer, T. A. , J. Owens , and P. G. Robinson . 2016. “The Acceptability of Healthcare: From Satisfaction to Trust.” Community Dental Health 33, no. 4: 242–251. 10.1922/CDH_3902Dyer10. 28537359

[msc70266-bib-0017] Elings, J. , G. van der Sluis , R. A. Goldbohm , et al. 2016. “Development of a Risk Stratification Model for Delayed Inpatient Recovery of Physical Activities in Patients Undergoing Total Hip Replacement.” Journal of Orthopaedic & Sports Physical Therapy 46, no. 3: 135–143. 10.2519/jospt.2016.6124. 26813752

[msc70266-bib-0018] Ethgen, O. , O. Bruyère , F. Richy , C. Dardennes , and J. Y. Reginster . 2004. “Health‐Related Quality of Life in Total Hip and Total Knee Arthroplasty. A Qualitative and Systematic Review of the Literature.” Journal of Bone and Joint Surgery 86, no. 5: 963–974. 10.2106/00004623-200405000-00012. 15118039

[msc70266-bib-0019] Fleurent‐Grégoire, C. , N. Burgess , D. I. McIsaac , et al. 2024. “Towards a Common Definition of Surgical Prehabilitation: A Scoping Review of Randomised Trials.” British Journal of Anaesthesia 133, no. 2: 305–315. 10.1016/j.bja.2024.02.035. PMC11282475 38677949

[msc70266-bib-0020] Fuchs, T. I. , C. Pfab , J. Kiselev , S. J. Schaller , C. Spies , and T. Rombey . 2024. “Barriers and Facilitators to the Implementation of Prehabilitation for Elderly Frail Patients Prior to Elective Surgery: A Qualitative Study With Healthcare Professionals.” BMC Health Services Research 24, no. 1: 536. 10.1186/s12913-024-10993-2. PMC11046874 38671446

[msc70266-bib-0021] Gale, N. K. , G. Heath , E. Cameron , S. Rashid , and S. Redwood . 2013. “Using the Framework Method for the Analysis of Qualitative Data in Multi‐Disciplinary Health Research.” BMC Medical Research Methodology 13, no. 1: 117. 10.1186/1471-2288-13-117. PMC3848812 24047204

[msc70266-bib-0022] Halsen, K. , O. E. Svinøy , G. Hilde , and B. Langhammer . 2023. “Better Before‐Better After: A Qualitative Phenomenology Study of Older Adults' Experiences With Prehabilitation Before Total Hip Replacement.” Orthopaedic Nursing 42, no. 6: 384–395. 10.1097/nor.0000000000000988. 37989159

[msc70266-bib-0023] Haughton, S. , K. Saravanan , L. A. McDonald , et al. 2025. “Acceptability of a Physiotherapy‐Led Intensive Prone Positioning Service in Intensive Care: A Qualitative Study With Multidisciplinary Clinicians.” Australian Critical Care 38, no. 3: 101162. 10.1016/j.aucc.2024.101162. 39892067

[msc70266-bib-0024] Hoogeboom, T. J. , J. J. Dronkers , E. H. Hulzebos , and N. L. van Meeteren . 2014. “Merits of Exercise Therapy Before and After Major Surgery.” Current Opinion in Anaesthesiology 27, no. 2: 161–166. 10.1097/aco.0000000000000062. PMC4072442 24500337

[msc70266-bib-0025] Husted, R. S. , T. Bandholm , M. S. Rathleff , A. Troelsen , and J. Kirk . 2020. “Perceived Facilitators and Barriers Among Physical Therapists and Orthopedic Surgeons to Pre‐Operative Home‐Based Exercise With One Exercise‐Only in Patients Eligible for Knee Replacement: A Qualitative Interview Study Nested in the QUADX‐1 Trial.” PLoS One 15, no. 10: e0241175. 10.1371/journal.pone.0241175. PMC7584251 33095777

[msc70266-bib-0026] James Lind Alliance . 2025. “Prehabilitation for Hip & Knee Replacement Surgery Priority Setting Partnership: Top 10 Priorities.” https://www.jla.nihr.ac.uk/priority‐setting‐partnerships/prehabilitation‐hip‐knee‐replacement‐surgery#tab‐top‐10‐priorities.

[msc70266-bib-0027] Karimijashni, M. , S. Yoo , K. Barnes , H. Lessard‐Dostie , T. Ramsay , and S. Poitras . 2024. “Prehabilitation in Patients at Risk of Poorer Outcomes Following Total Knee Arthroplasty: A Systematic Review.” Journal of Arthroplasty 40, no. 5: 1367–1376. 10.1016/j.arth.2024.10.132. 39510391

[msc70266-bib-0028] Khalid, T. , Y. Ben‐Shlomo , W. Bertram , et al. 2024. “Prehabilitation for Frail Patients Undergoing Hip and Knee Replacement in the UK: Joint PREP Feasibility Study for a Randomised Controlled Trial.” BMJ Open 14, no. 9: e084678. 10.1136/bmjopen-2024-084678. PMC11409239 39289012

[msc70266-bib-0029] Lan, R. H. , J. Yu , L. T. Samuel , M. A. Pappas , P. J. Brooks , and A. F. Kamath . 2020. “How Are We Measuring Cost‐Effectiveness in Total Joint Arthroplasty Studies? Systematic Review of the Literature.” Journal of Arthroplasty 35, no. 11: 3364–3374. 10.1016/j.arth.2020.06.046. 32680755

[msc70266-bib-0030] Longo, U. G. , S. De Salvatore , C. Rosati , et al. 2023. “The Impact of Preoperative Education on Knee and Hip Replacement: A Systematic Review.” Osteology 3, no. 3: 94–112. 10.3390/osteology3030010.

[msc70266-bib-0031] Macmillan Cancer Support . 2025. Prehabilitation for People With Cancer: Clinical and Implementation Guidelines. Prehabilitation resources for healthcare professionals. Macmillan Cancer Support. https://www.macmillan.org.uk/dfsmedia/1a6f23537f7f4519bb0cf14c45b2a629/22869‐10061/prehabilitation‐for‐people‐with‐cancer‐clinical‐and‐implementation‐guidelines.

[msc70266-bib-0032] Mohammadi, S. , H. Reid , W. Watson , et al. 2021. “A Qualitative Study on Prehabilitation Before Total Hip and Knee Arthroplasties: Integration of Patients’ and Clinicians’ Perspectives.” Disabilities 1, no. 4: 361–376. 10.3390/disabilities1040025.

[msc70266-bib-0033] Paynter, C. , C. McDonald , D. Story , and J. J. Francis . 2023. “Application of the Theoretical Framework of Acceptability in a Surgical Setting: Theoretical and Methodological Insights.” British Journal of Health Psychology 28, no. 4: 1153–1168. 10.1111/bjhp.12677. 37353989

[msc70266-bib-0034] Powell, R. , A. Davies , K. Rowlinson‐Groves , D. P. French , J. Moore , and Z. Merchant . 2023. “Acceptability of Prehabilitation for Cancer Surgery: A Multi‐Perspective Qualitative Investigation of Patient and 'Clinician' Experiences.” BMC Cancer 23, no. 1: 744. 10.1186/s12885-023-10986-0. PMC10416438 37568097

[msc70266-bib-0035] Punnoose, A. , L. S. Claydon‐Mueller , O. Weiss , J. Zhang , A. Rushton , and V. Khanduja . 2023. “Prehabilitation for Patients Undergoing Orthopedic Surgery: A Systematic Review and Meta‐analysis.” JAMA Network Open 6, no. 4: e238050. 10.1001/jamanetworkopen.2023.8050. PMC10102876 37052919

[msc70266-bib-0036] Raad, M. , R. M. Amin , J. M. El Abiad , V. Puvanesarajah , M. J. Best , and J. K. Oni . 2019. “Preoperative Patient Functional Status Is an Independent Predictor of Outcomes After Primary Total Hip Arthroplasty.” Orthopedics 42, no. 3: e326–e330. 10.3928/01477447-20190321-01. 30913294

[msc70266-bib-0037] Rossen, C. B. , A. M. Schmidt , M. S. Hansen , A.‐L. D. Wivelsted , P. Vedsted , and M. Gregersen . 2025. “Multicomponent Prehabilitation and Perioperative Care in Older Persons With Frailty Referred to Planned Orthopaedic Surgery: A Feasibility Study.” Aging Clinical and Experimental Research 37, no. 1: 316. 10.1007/s40520-025-03214-1. PMC12592306 41196467

[msc70266-bib-0038] Salaffi, F. , M. Carotti , A. Stancati , and W. Grassi . 2005. “Health‐Related Quality of Life in Older Adults With Symptomatic Hip and Knee Osteoarthritis: A Comparison With Matched Healthy Controls.” Aging Clinical and Experimental Research 17, no. 4: 255–263. 10.1007/bf03324607. 16285189

[msc70266-bib-0039] Schöne, C. , T. I. Fuchs , J. Kiselev , et al. 2025. “Facilitators and Barriers to Participation in Prehabilitation Prior to Orthopaedic Elective Surgery—A Qualitative Study With Elderly (pre‐)frail Patients.” BMC Geriatrics 25, no. 1: 845. 10.1186/s12877-025-06592-3. PMC12584233 41188708

[msc70266-bib-0040] Sekhon, M. , M. Cartwright , and J. J. Francis . 2017. “Acceptability of Healthcare Interventions: An Overview of Reviews and Development of a Theoretical Framework.” BMC Health Services Research 17, no. 1: 88. 10.1186/s12913-017-2031-8. PMC5267473 28126032

[msc70266-bib-0041] Smyth, E. , L. Brennan , R. Enright , et al. 2024. “The Acceptability of Exercise Prehabilitation Before Cancer Surgery Among Patients, Family Members and Health Professionals: A Mixed Methods Evaluation.” Supportive Care in Cancer 32, no. 6: 399. 10.1007/s00520-024-08574-4. PMC11142941 38819477

[msc70266-bib-0042] Sontag, A. F. , J. Kiselev , S. J. Schaller , C. Spies , and T. Rombey . 2024. “Facilitators and Barriers to the Implementation of Prehabilitation for Frail Patients Into Routine Health Care: A Realist Review.” BMC Health Services Research 24, no. 1: 192. 10.1186/s12913-024-10665-1. PMC10863196 38350947

[msc70266-bib-0043] Tong, A. , P. Sainsbury , and J. Craig . 2007. “Consolidated Criteria for Reporting Qualitative Research (COREQ): A 32‐Item Checklist for Interviews and Focus Groups.” International Journal for Quality in Health Care 19, no. 6: 349–357. 10.1093/intqhc/mzm042. 17872937

[msc70266-bib-0044] Vaishya, R. , R. P. Mahajan , and A. Sibal . 2024. “Prehabilitation: A Proactive Approach to Enhancing Outcomes in Joint Replacement Surgery.” Journal of Clinical Orthopaedics and Trauma 51: 102398. 10.1016/j.jcot.2024.102398. PMC10998185 38585383

[msc70266-bib-0045] van der Velde, M. , M. van der Leeden , E. Geleijn , C. Veenhof , and K. Valkenet . 2023. “What Moves Patients to Participate in Prehabilitation Before Major Surgery? A Mixed Methods Systematic Review.” International Journal of Behavioral Nutrition and Physical Activity 20, no. 1: 75. 10.1186/s12966-023-01474-6. PMC10286498 37344902

[msc70266-bib-0046] Vervullens, S. , L. Meert , I. Baert , et al. 2023. “Prehabilitation Before Total Knee Arthroplasty: A Systematic Review on the Use and Efficacy of Stratified Care.” Annals of Physical and Rehabilitation Medicine 66, no. 4: 101705. 10.1016/j.rehab.2022.101705. 36115573

[msc70266-bib-0047] Wainwright, T. W. , M. Gill , D. A. McDonald , et al. 2020. “Consensus Statement for Perioperative Care in Total Hip Replacement and Total Knee Replacement Surgery: Enhanced Recovery After Surgery (ERAS R) Society Recommendations.” Acta Orthopaedica 91, no. 1: 3–19. 10.1080/17453674.2019.1683790. PMC7006728 31663402

