# Supplementary material for: ‘Prehabilitation Should Adapt to the Person, not the Other Way Around’: A Qualitative Study Exploring Stakeholder Perceptions of Prehabilitation for Total Hip and Knee Arthroplasty
Source: Musculoskeletal Care. 2026 Oct 8;24(4):e70266. doi: 10.1002/msc.70266 (PMC13648159; doi:10.1002/msc.70266)
Supplement: Supplementary file 1 [file MSC-24-e70266-s001.pdf]

Supplementary Information

"Prehab should adapt to the person, not the other way around": A qualitative study exploring stakeholder perceptions of prehabilitation for total hip and knee arthroplasty.

Table of contents

eTable 1. Semi-structured interview guides informed by the TDF and TFA 2

eTable 2. Explanation of the 14 domains of the Theoretical Domains Framework[1]......................6

eTable 3. Explanation of the 7 original domains of the Theoretical Framework of Acceptability[2] and additional domain suggested by Paynter et al.[3] 7

eTable 4. Consolidated criteria for reporting qualitative studies (COREQ): 32-item checklist[4] 8

eTable 5. Sub-themes and example quotes for Concept 1: 'Understanding and beliefs around prehabilitation' 12

eTable 6. Sub-themes and example quotes for Concept 2: 'Barriers and enablers influencing implementation of prehabilitation in the joint arthroplasty population' 15

eTable 7. Sub-themes and example quotes for Concept 3: 'Strategies for successful implementation of prehabilitation' 22

References 25

eTable 1. Semi-structured interview guides informed by the TDF and TFA

Healthcare professional interview guide

**Background (5 mins)**	
• What is your profession?<br>• How long have you been working with patients receiving total hip or knee arthroplasty surgery? And what is your involvement in the care journey?<br>• What is your highest level of qualification?	
**Acceptability of prehabilitation (10 mins)**	**TDF/TFA domain**
• How would you define prehab?<br>• What is your understanding of the goals or benefits of prehab?<br>• Do you think it's important/makes sense to offer patients prehabilitation prior to hip and knee arthroplasty? Why?	*TDF: Knowledge, Skills*<br>*TFA: Intervention coherence; Affective attitude*
• What do you think constitutes a poor surgical outcome for total hip or knee arthroplasty? What would be the indicators?<br>• Do you think prehab could change this at all? How would you measure success of prehab?	*TDF: Beliefs about consequences*<br>*TFA: Perceived effectiveness*
• Do you think there are any ethical issues with offering prehab? *Prompt: in what ways do you think offering prehab before surgery is fair or not fair?*<br>• Do you think there are any consequences or negative effects? *Prompts: To the patients, yourself, or the healthcare system?*	*TDF: Beliefs about consequences*<br>*TFA: Ethicality; Safety and Risk*
• Do you think all patients awaiting a hip or knee replacement would benefit from prehabilitation? Are there some groups that are more likely than others? Which ones and why?	*TDF: Beliefs about consequences; Optimism*<br>*TFA: Perceived effectiveness, Ethicality*
**Factors influencing referral and delivery (15 mins)**	**TDF/TFA domain**
• Do you discuss prehab with all patients? *Prompts: What proportion? Why some and not others?*<br>• What type of information do you discuss with patients about prehab? How confident are you to provide this information?<br>• When/at what stage do you usually provide this information, or when do you think it's important to discuss the concept of prehabilitation?	*TDF: Memory, attention and processes; Beliefs about capabilities*<br>*TFA: Self-efficacy*
• (For surgeons and anaesthetists) Do you refer patients to a formal prehabilitation program prior to their surgery? Why or why not?<br>• (For surgeons and anaesthetists) Are there any barriers/costs to you discussing prehab with your patients or referring patients to prehabilitation? What would/does make it easier for you to discuss/refer patients?	*TDF: Intentions; Behaviour regulation; Environmental context and resources*<br>*TFA: Opportunity costs*
• (For physiotherapists) Are there any physical or environmental barriers or costs to you (or the health service) delivering prehabilitation to patients? What would/does make it easier for you to deliver a prehab program?	*TDF: Intentions; Behaviour regulation; Environmental context and resources*<br>*TFA: Burden, Opportunity costs*
• Who do you think is the most appropriate person/profession to discuss prehab with patients?	*TDF: Social/professional role and identity*

• To what extent do you think providing information about prehabilitation is part of your role?<br>• Do you think your colleagues have similar approaches to discussing prehab or referring patients to prehab?	
• (Any profession) In your opinion, are you aware of any factors that influence a patient's decision/ability to complete prehab or not? What are the main reasons people do or don't accept prehab?	*COM-B: Beliefs about capabilities; Environmental context and resources*
**Perspectives on care models and content (10 mins)**	**TDF/TFA domain**
• What do you think are the most important elements to include in a prehab program? *Prompts: Education – any particular modules? Multi-disciplinary team– who should be involved?*<br>• When do you think is the best time to start prehab?	*COM-B: Knowledge; Skills*<br>*TFA: Affective attitude*
• What do you think is the best mode of delivery for a prehab program?<br>• How would you feel if there was an online option for prehabilitation program delivery? *Prompts: Any concerns? Any previous experience with online programs?*	*TDF: Environmental context and resources*<br>*TFA: Perceived effectiveness*
• What skills/resources are needed to promote/deliver a prehab program? Are there any skills you would like to improve in this area?<br>• To what extent do you feel you have the necessary resources to (promote/deliver) the program you have described?	*TDF: Knowledge; Skills; Environmental context and resources*
**Is there anything else you would like to add about prehabilitation that we haven't already covered?**	

Patient interview guide

Background (3 mins)	
• Have you already had your operation or are you still awaiting surgery? Which joint?<br>• How long have you been/were you on the waitlist?	
**Acceptability of prehabilitation (10 mins)**	**TDF/TFA domain**
• Before this study, had you heard of prehabilitation before? *Prompts: Where/who provided this information?*<br>• What is your understanding of prehabilitation? *Prompts: How does it work? Why is it recommended?*	*TDF: Knowledge*<br>*TFA: Perceived effectiveness; Intervention coherence*
• How do you feel about the concept of prehabilitation? Does the idea of prehab raise any emotions for you? *Prompts: Can be positive or negative*	*TDF: Emotions*<br>*TFA: Affective attitude*
• Were you, or have you been, offered prehabilitation prior to your surgery? Who referred you?<br>• *As appropriate:* were the services offered to you suitable and acceptable/meet your needs? Why/why not?	*TDF: Environmental context and resources*<br>*TFA: Burden*
• What would be a poor surgical outcome in your opinion?<br>• (If completed prehab) Do you think prehab was effective for you? *Prompts: Do you feel like completing prehab prior to surgery changed your outcomes after the surgery compared to if you had not done prehab?*<br>• (If did not complete prehab) Do you think there are any benefits of participating in prehab? What outcomes would you expect to see?<br>• Do you think there are any risks involved in participating in prehab?	*TDF: Belief about consequences*<br>*TFA: Perceived effectiveness, Perceived safety*
**Factors influencing uptake or access (15 mins)**	**TDF/TFA domain**
• Did you do/have you done, or do you plan on doing, anything specific in preparation for your surgery?<br>• Was there anything in particular that helped you prepare for surgery? Anything that prevented you (or might stop you in the future)?	*TDF: Intentions; Goals; Reinforcement*
• Did you/have you receive/d any information about prehab or how to prepare prior to your surgery? *Prompts: Did you feel you received enough information? Any additional information you would've liked?*<br>• (If yes) From whom/where did you receive this information and when?<br>• (If no) Would you have liked to receive more information? Specifically, what would you like to have been more informed about?	*TDF: Environmental context and resources*
(If offered prehab):<br>• Did you choose to participate in a prehabilitation program?<br>• *(if yes)* How confident were you that you could complete the prehab program?<br>• What were the biggest factors that influenced your decision/ability to complete prehab or not?<br>• Can you identify any barriers to participating in prehab?<br>• How easy was it to complete the prehab program/how much effort did it take (session length, finding the time etc)? Was there anything you had to give up to engage in prehab?<br><br>*(If not offered prehab):*	*TDF: Behaviour regulation, Beliefs about capabilities; Intentions*<br>*TFA: Burden, Opportunity costs, Self-efficacy*

• If you were offered prehab before your surgery, how likely do you think you would be to participate? What factors would influence that decision?<br>• What are the barriers? What would have to change to enable you to participate in a prehabilitation program?	
• How do you think your friends/family would influence/influenced your decision to participate in prehabilitation or not? What about healthcare professionals?<br>• Is there anything you feel you would need/would have needed to help you participate in prehab prior to your surgery? *Prompts: Support or skills, physical resources*	*TDF: Social influences; Environmental context and resources; Skills*
**Perspectives on care models and content (10 mins)**	**TDF/TFA domain**
• What do you think are the most important elements to include in a prehab program? *Prompts: Education – any particular topics to cover? Exercise – what type? Who should be involved – dietetics, psychology, physiotherapy, social work?*<br>*(For patients who have had surgery):*<br>• Looking back, is there anything you wish you had known/had the opportunity to do prior to surgery that you think should be included in prehab?	*TDF: Knowledge; Skills*<br>*TFA: Affective Attitude*
• Who do you think is best positioned to deliver information about prehab? When/how would you like to receive this information?	*TDF: Social influences*
• What do you think is the best way to deliver a prehab program?<br>• How would you feel if there was an online option for prehabilitation program delivery? *Prompts: Any concerns? Any previous experience with online programs? Do you think you would engage with an online program?*<br>• Do you think everyone should be offered prehab? Do you think there are any ethical issues with offering prehab? *Prompt: In what ways do you think prehab before surgery is fair or not fair?*	*TDF: Environmental context and resources*<br>*TFA: Perceived Effectiveness*<br>*Ethicality*
**Is there anything else you would like to add about prehabilitation that we haven't already covered?**	

eTable 2. Explanation of the 14 domains of the Theoretical Domains Framework[1]

Domain	Explanation
**1. Knowledge**	An awareness of the existence of something (e.g. Evidence, guidelines)
**2. Skills**	An ability or proficiency acquired through practice (e.g. New or preestablished skills may be required to participate in the intervention)
**3. Social/professional role and identity**	A coherent set of behaviours and displayed personal qualities of an individual in a social or work setting
**4. Beliefs about capabilities**	Acceptance of the truth, reality or validity about an ability, talent, or facility that a person can put to constructive use (e.g. Self-confidence, empowerment)
**5. Optimism**	The confidence that things will happen for the best or that desired goals will be attained
**6. Beliefs about consequences**	Acceptance of the truth, reality or validity about outcomes of a behaviour in a given situation (e.g. Outcomes that are assumed to be associated with future or intended behaviour)
**7. Reinforcement**	Increasing the probability of a response by arranging a dependent relationship, or contingency, between the response and a given stimulus (e.g. Rewards, incentives, punishments)
**8. Intentions**	A conscious decision to perform a behaviour or a resolve to act in a certain way
**9. Goals**	Mental representations of outcomes or end states that an individual wants to achieve (e.g. Behavioural targets that are measurable, achievable and realistic)
**10. Memory, attention, and decision processes**	The ability to retain information, focus selectively on aspects of the environment and choose between two or more alternatives
**11. Environmental context and resources**	Any circumstance of a person's situation or environment that discourages or encourages the development of skills and abilities, independence, social competence and adaptive behaviour (e.g. staffing levels, time, equipment, organizational culture)
**12. Social influences**	Interpersonal processes than can cause individuals to change their thoughts, feelings or behaviours (e.g. Patients, families, group norms)
**13. Emotion**	A complex reaction pattern, involving experiential, behavioural, and physiological elements, but which the individual attempts to deal with a personally significant matter or event (e.g. work stress or patient anxiety that can help or hinder execution of a behaviour)
**14. Behavioural regulation**	Anything aimed at managing or changing objectively observed or measured actions (e.g. Practical strategies like action planning that facilitate or hinder uptake of a behaviour)

eTable 3. Explanation of the 7 original domains of the Theoretical Framework of Acceptability[2] and additional domain suggested by Paynter et al.[3]

Domain	Explanation
**1. Affective attitude**	How an individual feels about the intervention
**2. Burden**	Perceived amount of effort required to participate in the intervention
**3. Ethicality**	Extent to which the intervention has good fit an individual's value system
**4. Intervention coherence**	Extent to which the participant understands the intervention and how it works
**5. Opportunity costs and gains**	Extent to which benefits, profits, or values must be given up, or are gained, by engaging in the intervention
**6. Perceived effectiveness**	Extent to which intervention is perceived as likely to achieve its purpose
**7. Self-efficacy**	Participant's confidence that they can perform the behaviour required to participate in the intervention
**8. Perceived safety and risk**	Perceived, or experienced, threats and harms or feelings of safety

eTable 4. Consolidated criteria for reporting qualitative studies (COREQ): 32-item checklist[4]

No. Item	Guide questions/description	Response
**Domain 1: Research team and reflexivity**		
Personal characteristics		
**1. Interviewer/ facilitator**	Which author/s conducted the interview or focus group?	Researcher N.B undertook all interviews
**2. Credentials**	What were the researcher's credentials? E.g. PhD, MD	N.B: Doctor of Physiotherapy, Bachelor of Biotechnology and Medical Research, PhD candidate in physiotherapy<br>E.W: Masters of Public Health<br>M.G: Bachelor of Physiotherapy, Masters of Public Health, PhD
**3. Occupation**	What was their occupation at the time of the study?	N.B: Clinical physiotherapist and PhD candidate in physiotherapy<br>E.W: Clinical research nurse in orthopaedic surgery and PhD candidate in orthopaedic surgery<br>M.G: Clinical researcher / Implementation Scientist
**4. Gender**	Was the researcher male or female?	Female (N.B, E.W, M.G)
**5. Experience and training**	What experience or training did the researcher have?	M.G is a trained academic with 10 years experience in conducting qualitative research and implementation science<br>N.B and E.W both have >3 years of experience in conducting and analysing qualitative research.<br>The expertise of the other authors on this paper spans qualitative, quantitative and mixed methods research in diverse clinical areas, including orthopedics, digital health, prehabilitation and rehabilitation, and nursing.
Relationship with participants		
**6. Relationship established**	Was a relationship established prior to study commencement?	Whilst the researcher undertaking the interviews (N.B) was employed at the study site it is important to note that they did not have a current or prior clinical relationship with any study participants receiving hospital or community care. The researcher had working relationships with some of the clinicians interviewed.
**7. Participant knowledge of the interviewer**	What did the participants know about the researcher? e.g. personal	The researcher N.B verbally explained their role (physiotherapist with clinical and research expertise, PhD student), affiliation with the

	goals, reasons for doing the research	University and purpose of the research prior to the commencement of the interviews.
**8. Interviewer characteristics**	What characteristics were reported about the inter viewer/facilitator? e.g. Bias, assumptions, reasons and interests in the research topic	Participants were informed that the researcher conducting the interview was interested in the pre-operative patient and clinician experience as a physiotherapist. Participants were also told the researcher was currently undertaking their PhD through the university.<br>The researcher conducting interviews was aware that she had developed various preconceptions about potential barriers and facilitators to engagement with prehabilitation through her clinical physiotherapy work and wider reading. To minimise the impact of these preconceptions on her interpretation of the data, she employed various strategies to enhance confirmability including field notes to record reflections on aspects such as her preconceptions, dual position as a researcher and health professional, and challenges encountered during the analysis process. The data were analysed independently by two researchers to identify themes. The second researcher was of a different clinical background (nursing). Themes were reviewed, challenges and reorganised through robust discussions with all 3 researchers.
**Domain 2: study design**		
Theoretical framework		
**9. Methodological orientation and Theory**	What methodological orientation was stated to underpin the study? e.g. grounded theory, discourse analysis, ethnography, phenomenology, content analysis	Inductive thematic analysis followed by deductive content analysis using the theoretical domains framework (TDF) and theoretical framework of acceptability (TFA) underpinned this study. The methodology was guided by the Framework Analysis.
Participant selection		
**10. Sampling**	How were participants selected? e.g. purposive, convenience, consecutive, snowball	Purposive sampling was undertaken
**11. Method of approach**	How were participants approached? e.g. face-to-face, telephone, mail, email	Patient participants were approached in person (at pre-admission clinic or post-operative rehabilitation appointment) or by telephone. Clinician participants were approached in person or by email.

**12. Sample size**	How many participants were in the study?	N=30
**13. Non-participation**	How many people refused to participate or dropped out? Reasons?	9 people declined (n=3 clinicians, n=6 patients) and 8 were unable to be contacted. Reasons for declining included time availability (n=3 clinicians and n=3 patients), not interested (n=2 patients) and medically unwell (n=1 patient).
Setting		
**14. Setting of data collection**	Where was the data collected? e.g. home, clinic, workplace	Data were collected either online via Microsoft Teams (participants could be at home or in the hospital setting) or in a private meeting room in the physiotherapy outpatient department.
**15. Presence of non-participants**	Was anyone else present besides the participants and researchers?	An interpreter was present online for 3 interviews. For 3 interviews, family members/carers attended with participants but did not participate in the interview.
**16. Description of sample**	What are the important characteristics of the sample? e.g. demographic data, date	Participant characteristics are presented in Table 1.
Data collection		
**17. Interview guide**	Were questions, prompts, guides provided by the authors? Was it pilot tested?	Guiding questions for interviews were guided by the domains/constructs of TDF and TFA. The interview questions were reviewed and modified by other members of the research team (M.G and L.E) with extensive experience in qualitative research.
**18. Repeat interviews**	Were repeat interviews carried out? If yes, how many?	No repeat interviews were conducted.
**19. Audio/visual recording**	Did the research use audio or visual recording to collect the data?	All interviews were audio recorded and transcribed verbatim aided by Microsoft Teams transcription (checked for accuracy by N.B).
**20. Field notes**	Were field notes made during and/or after the interview or focus group?	Field notes were taken by the researcher (N.B) during all data collection.
**21. Duration**	What was the duration of the inter views or focus group?	Interviews averaged approximately 40 minutes.
**22. Data saturation**	Was data saturation discussed?	Yes, data collection was ceased after 30 interviews as no new themes had emerged.
**23. Transcripts returned**	Were transcripts returned to participants for comment and/or correction?	Transcripts were not returned to participants following the interview unless requested. No participants requested the transcript for review.
**Domain 3: analysis and findings**		
Data analysis		

**24. Number of data coders**	How many data coders coded the data?	Two researchers coded the data (N.B and E.W) with arbitration by a third researcher (M.G).
**25. Description of the coding tree**	Did authors provide a description of the coding tree?	Codes were first inductively coded by researchers, followed by deductive coding based on the TDF and TFA.
**26. Derivation of themes**	Were themes identified in advance or derived from the data?	Themes were first derived from the data, followed by mapping of themes to established frameworks.
**27. Software**	What software, if applicable, was used to manage the data?	Qualitative data were managed using NVivo version 12.
**28. Participant checking**	Did participants provide feedback on the findings?	Participants were not provided an opportunity to give feedback on the research findings.
Reporting		
**29. Quotations presented**	Were participant quotations presented to illustrate the themes/findings? Was each quotation identified? e.g. participant number	Representative verbatim participant quotations are presented throughout the Results section.
**30. Data and findings consistent**	Was there consistency between the data presented and the findings?	Researchers have demonstrated consistency between data presented and findings through representations in written text, participant quotations and tables/figures.
**31. Clarity of major themes**	Were major themes clearly presented in the findings?	Three overarching themes were presented in the findings with multiple sub-themes underpinning each. Tables 2 and 3 include the number of participants who expressed each sub-theme, providing clarity on how frequently a sub-theme was discussed in interviews.
**32. Clarity of minor themes**	Is there a description of diverse cases or discussion of minor themes?	Diverse participant experiences were represented in the results and conflicting findings between participant groups were discussed where present.

eTable 5. Sub-themes and example quotes for Concept 1: 'Understanding and beliefs around prehabilitation'

Item	Sub-theme	Example quotes
		**1. Knowledge of and attitude towards prehabilitation**
**1.1**	There is varying awareness/knowledge of prehabilitation amongst both healthcare professionals and patients, which can impact acceptability (B/E)	• So my understanding of prehab is sort of like you wouldn't run a marathon without training for it, so you wouldn't have a joint replacement without sort of training for it. So that's my understanding is it's sort of like training for surgery in a way from a physical and mental point of view. *(P9 – physiotherapist)*<br>• I feel like I don't know too much about prehabilitation, to be honest with you. *(P21 – surgical liaison nurse)*<br>• No, I have not heard of prehabilitation. I know about rehab… *(P29 – patient, male, 80, pre-TKA, CALD background)*<br>• But often when I do sort of explain to them like why it's important, what we offer…just giving them that education can sort of sort of change their mind around prehab. *(P9 – physiotherapist)*<br>• I think just having that advice and knowledge on the benefits would actually get me in the right frame of mind to do things. *(P27 – patient, male, 75, pre-THA, CALD background)*<br>• I think prehabilitation should always factor in patient education, it should be considered a minimum requirement…we can tell the patient to do what we think is best, but if they don't really understand and believe why that is necessary for a good outcome, then they won't do it. *(P20 – surgeon)*<br>• I don't know anything about [prehab]. Someone would have had to do a bit of convincing to get me to do the prehab. *(P8 – patient, male, 72, post-TKA)*
**1.2**	Prehabilitation is generally viewed as a beneficial, low risk intervention (E)	• I think if I had had that available, I think it's a good thing. Because it just makes you more aware of what, like what the procedure will be, what the outcomes can be. You know how you can actually help yourself prepare for that surgery. I think all those things are really good. *(P3 – patient, female, 68, post-THA)*<br>• I feel very positively about prehab. It gets you up and moving and it shows you that you can still do things. You know, because when you're in pain, you tend to just sit all the time, even getting up and getting down hurts. But coming here and doing things, I felt that I got a bit more flexible and strong type thing. I mean, it still hurt, but I knew I could do things still. *(P6 – patient, female, 78, post-THA)*<br>• The short time I spent [doing prehab] has made a difference. In terms of lack of painkillers and those medications and things, it's much better. And my confidence in what I can do without making things worse. *(P14 – patient, female, 77, pre-TKA)*<br>• There is really no risk in doing a prehab programme beforehand - all you can do is be better prepared for surgery. *(P4 – physiotherapist)*

**1.3**	Difficulty exists in clarifying prehabilitation success and goals in this population (B)<br>- Hard to define<br>- Unclear measures of success<br>- Lack of strong evidence	• I don't really know what [prehabilitation] success is. It's our best guess, we don't have an objective way of working out who will benefit and who won't. Patient-reported outcomes is probably the most important, but it's not a clean measurement, there's other things that affect a PROM. *(P12 –anaesthetist)*<br>• My overarching question is how do we know we're making a difference to these people and are we doing the right thing? *(P23 – physiotherapist)*<br>• I really don't know what the answer is there because it's not obvious. It's so hard, like it's so obvious in oncology where you've got survivorship as the most obvious outcome. *(P11 – physiotherapist)*<br>• I think you have to limit the definition to the end of the preoperative phase because then anything could happen after that which is not the fault of prehabilitation right? *(P20 – anaesthetist)*<br>• From my understanding, I think the evidence for prehab, you know mitigating or improving postoperative outcomes is a little bit limited at least in terms of randomised control evidence. *(P10 – anaesthetist)*<br>• I've had cases where they do prehab then they go "I don't want to have surgery anymore". That really shouldn't be happening. Because then you've got this blurred line between prehab and conservative management, and what the goal is. *(P2 – physiotherapist)*<br>• But it's all messy cause the pathway is so unclear. We need to differentiate and have really clear goals and objectives of each. *(P23 – physiotherapist)*
**1.4**	For those offered prehabilitation, there was a sense of gratitude (E)	• I was grateful when they offered me this prehab because I felt that people were interested in my own well-being, they wanted to help me get through this surgery. I was grateful for this chance to participate in the program. *(P27 – patient, male, 75, pre-THA, CALD background)*<br>• I didn't know where to start. I've got no idea what to do before the surgery. But then a couple of months later I got a call from the physio asking if I wanted to do this program at the hospital to prepare for surgery. And it felt like a relief, because I didn't have a clue what to do to prepare myself. *(P30 – patient, male, 64, pre-THA, CALD background)*<br>• The prehab gave me a great opportunity, which was really helpful for me, to talk to other people. I'm so glad I have that opportunity *(P5 – patient, female, 67, post-THA)*
	**2. Perceived goals and benefits of prehabilitation**	
**2.1**	Participating in prehabilitation improves post-operative recovery (E)	• I've suffered a bit after the operation, trying to do the rehab, my leg muscles were paining something shocking after the physio, and I think it was because I was a bit inactive beforehand. That's what I think prehab is, just getting in and doing something would improve the recovery. Not that I had it, but I think now after experiencing the post-op physio, that it probably would've been helpful. *(P8 – patient, male, 72, post-TKA)*<br>• Certainly [prehab] is very important. Someone who is prepared mentally and physically will recover much better, faster. *(P28 – patient, male, 74, pre-TKA, CALD background)*

		• I think there's definitely benefits, especially if you're going to go straight into surgery afterwards, because then your recovery time would be faster and rebuilding that muscle after surgery would be faster also. *(P24 – patient, female, 52, post-THA)*<br>• I would just think that your recovery would be harder and longer if you weren't trying to keep the muscles going in that beforehand time. *(P13 – patient, female, 66, pre-TKA)*<br>• It makes the transition a lot easier. They're so much more, knowing they've got to do these exercises, they've met the people in the team before. It just makes all the difference, particularly if they have lower health literacy. *(P9 – physiotherapist)*<br>• Ideally, it impacts the patient's immediate hospital experience but also it means that their trajectory of recovery postoperative is faster and easier. *(P23 – physiotherapist)*<br>• I want to recover better and have smoother recovery. I want to return to my normal life. Get back to work. Anything that will help make recovery faster, any preparation, I will do. *(P28 – patient, male, 74, pre-TKA, CALD background)*
**2.2**	There is a sense of control and empowerment through participating in prehabilitation (E)	• You put everything on the surgeon's shoulders that you will come through it. So I think [prehabilitation] just gave you the ability to know that you can get there sort of thing. You know you've done your bit. *(P6 – patient, female, 78, post-THA)*<br>• If people tell you what to do before your operation you feel like you've got a purpose *(P13 – patient, female, 66, pre-TKA*<br>• They are more empowered on how they can help themselves and what they need to do, post operatively to get back to life. *(P23 – physiotherapist)*
**2.3**	Belief that participating in prehabilitation has the potential to reduce resource use and clinician burden post-operatively (E)	• If I've got someone in the post-operative group that has done prehab, I can get them started and get them going quicker and easier and then focus on the people that need it more…there's less burden on me. *(P1 – physiotherapist)*<br>• Well, the only thing I would suggest as a possible benefit is that…You may get to the point of is surgery actually necessary? Because sometimes I reckon prehabilitation can actually improve your situation to the point where maybe you don't need surgery anymore. *(P8 – patient, male, 72, post-TKA)*<br>• The health service benefits, or the insurer if it's private, because usually those people who have done prehab get going more quickly, so they could potentially get out of hospital more quickly, less need for rehab after, physio etcetera. (P17 – surgeon)

= Voiced by both healthcare professionals and patients

= Voiced by healthcare professionals only

= Voiced by patients only

Abbreviations: B/E = combined barrier and enabler; B= barrier; E= enabler; HCP = healthcare

eTable 6. Sub-themes and example quotes for Concept 2: 'Barriers and enablers influencing implementation of prehabilitation in the joint arthroplasty population'

Item	Sub-theme	Example quotes
		**3. Patient-related implementation factors**
**3.1**	Physical capability influences ability to participate in prehabilitation (B/E)	• The obvious one for the demotivating and decline of prehab is that they're in too much pain...They're happy to exercise as long as the pain's tolerable. As soon as the pain's intolerable, they don't want to move. (P11 – physiotherapist)<br>• I think the only thing is you know, like I said before, once the pain really kicked in, once my hip really started playing up, I just hit a pinnacle where I just could not keep doing the prehab exercises. *(P7 – patient, male, 56, post-THA)*<br>• I think there's a group of patients that are just too sore to participate. It's just no way, regardless of our wishes. From a pain point of view there's no way they're gonna be able to do much. *(P22 – surgeon)*<br>• I was in a lot of pain. I don't know if I would've actually been able to do any prehab if it was offered. *(P25 – patient, female, 79, post-TKA, CALD background)*<br>• I can't do a lot right now while I am waiting for the surgery due to the physical pain. *(P30 - patient, male, 64, pre-THA, CALD background)*<br>• I've heard of people doing a bit of extra work before surgery but I counted that out because how bad my knees were. *(P8 – patient, male, 72, post-TKA)*
**3.2**	Previous experience with exercise or surgery influences uptake (B/E)	• I just wanted to keep moving, knowing that if I just sat there and did nothing I wasn't helping myself. Because I was also doing walking in the warm pool. I'd been doing that since 2020 to try and fix my back and everything so I know that it did benefit me. *(P6 – patient, female, 72, post-THA)*<br>• There's also the element that you've done [knee surgery] before and you know what to expect so that's a big part of it as well. *(P16 – patient)*<br>• Previous experience is a classic barrier, 'I've tried Physio, didn't work'. *(P19 – surgeon)*<br>• I think with some patients they're worried about getting injured or that they won't be able to do it. They haven't got that experience, they're not really sure what it's all about it so they avoid it. *(P2 – physiotherapist)*
**3.3**	Patient belief system/mindset impacts uptake (B/E)	• I'll do anything to get myself right. I've got a goal. That comes from your own mental attitude. *(P3 – patient, female, 68, post-THA)*<br>• People's belief system about well, I have arthritis, therefore I shouldn't exercise is also a limiting factor. *(P4 – physiotherapist)*

		• I don't know whether I would have done prehab or not if it was offered to me, you know. Someone would have had to do a bit of convincing to get me to do the prehab. *(P8 – patient, male, 72, post-TKA)*<br>• I don't know what it is, but some people just want it to happen to them. You know, not taking responsibility for themselves...that external locus of control. *(P18 – surgeon)*<br>• There's the ones that feel like [prehab] is not going to do anything or change anything. *(P1 – physiotherapist)*<br>• Often their belief is I'm having the joint replacement, particularly if they like have been told by the surgeon, "it's going to be within a few months or you know it's coming up", then a common phrase I get from patients is, "oh, I'll just wait for the joint replacement and then I'll do exercise afterwards." *(P9 – physiotherapist)*<br>• But I was worried about the exercise, I didn't want to do anything that made it worse. I didn't know if it was safe for me. *(P26 – patient, female, 66, post-TKA, CALD background)*
**3.4**	A individuals' situation/background can impact acceptability and uptake:<br>• CALD background<br>• Travel<br>• Financial<br>• Employment/ availability<br>• Social roles (B/E)	• Because of the distance between where I live and the hospital, and because I am still working, it was too hard to come in to do the prehab. *(P7 – patient, male, 56, post-THA)*<br>• Transport is an issue like if they have that much disability and they don't have someone to bring them in to do exercise. *(P1 – physiotherapist)*<br>• But for the patient who is culturally, linguistically diverse and can't come into clinic, there's then a significant limitation on what we can offer them. Because we don't have translated resources, we don't have an easily accessible portal. *(P23 – physiotherapist)*<br>• I was concerned about coming to the physio afterwards because of the language barrier. I needed my daughter with me because I don't understand. *(P25 – patient, female, 79, post-TKA, CALD background)*<br>• My priority in life is caring for my daughter and my wife. So that would take priority for me. So for me to do something like that [prehabilitation] it would have to fit in with my other priorities. *(P16 – patient, male, 71, post-TKA)*
**3.5**	Social environment can impact uptake (E)<br>• Family and friends<br>• Peer support/role models	• I talked to my friends [before the surgery], because I trust them, they are like me. *(P29 – patient, male, 80, pre-TKA, CALD background)*<br>• [My family] were all for [prehabilitation]. They encouraged it. Although I would have done it regardless, sort of thing. But yeah, they're all very happy that I was doing something. *(P6 – patient, female, 72, post-THA)*<br>• But also the peer support and learning that comes with, you know, people asking questions. And other people hearing those questions being answered. *(P1 – physiotherapist)*<br>• When you can get that information from someone that's just like you, that has gone through the same things, that is really powerful… I just felt like sometimes I really found it difficult to talk to my family about it, even though they could see I was struggling because I didn't want to worry them. And so just kind of having that, somewhere to go, was really beneficial. *(P5 – patient, female, 67, post-THA)*

**3.6**	Implicit trust in the care teams recommendations promotes uptake (E)	• If I was given advice on what to do to prepare, I would just more than likely trust in them because they are the professionals… You sort of just have to trust them, don't you? *(P13 – patient, female, 66, pre-TKA)*<br>• And once you've been told by a surgeon, then it's like the surgeon told me I have to do it. I trust them. But if it's somebody not that high up on the hierarchy, I might not be as likely to listen. *(P24 – patient, female, 52, post-THA)*<br>• I trust doctors. Any advice they gave me, I do. *(P29 – patient, male, 80, pre-TKA, CALD background)*<br>• And I take their advice. So I do have respect for the health professionals, whether it be doctors, physios...I take advice from experienced people. *(P8 – patient, male, 72, post-TKA)*
	**4. Healthcare professional-related implementation factors**	
**4.1**	Healthcare professionals' confidence in discussing/delivering prehabilitation is linked with knowledge and experience (B/E)	• Having experience working in the different areas [inpatients, outpatients and rehab], I think gives me that confidence. *(P1 – physiotherapist)*<br>• I think it's so important for clinicians to have an understanding of the surgeries definitely and also understanding OA management across the spectrum. *(P2 – physiotherapist)*<br>• I feel like a non-expert in prehab so I don't really feel confident to have that conversation. *(P10 – anaesthetist)*<br>• I have worked here for a really long time and I know the setting, and I know the team. I find it really easy to have those conversations around prehab. *(P11 – physiotherapist)*
**4.2**	Lack of clarity exists regarding professional roles and involvement in prehabilitation pathway, and how the care team works together (B)	• I don't know whether [the surgeons] talk about prehab at all. I have very little contact with [the multi-disciplinary team]. *(P2 – physiotherapist)*<br>• Anesthetists can definitely have a role in promoting prehab. But do we feel empowered to suggest prehab? ... Is it OK for me to them about prehabilitation? Or does the surgeon just want them operated on? *(P20 – anaesthetist)*<br>• The fact that I don't know if surgeons are even discussing [prehab] tells you, and I sit next to them once a week. *(P23 – physiotherapist)*<br>• I don't think people in the wider team have the same approach to discussing prehab, no. (P4 – physiotherapist)<br>• I think most of them, my colleagues, the ones I work with, have an understanding of prehab. But I don't think it's necessarily standardised among surgeons. *(P19 – surgeon)*<br>• I don't think people in the wider team have the same approach to discussing prehab, no. I mean from a physio point of view, I think we do especially in our role and some surgeons I know have highlighted it. But I feel that when they go to that surgeon appointment and they're consenting for surgery, it is not high on the surgeon's priority or the patients priority list to be talking about that. *(P4 – physiotherapist)*
**4.3**	Time is a barrier to discussing/delivering prehabilitation (B)	• We have 10 or 15 minute slots. There's no way you can fit it all in. *(P22 – surgeon)*<br>• There's just so many things that you need to do, and it's cognitively very taxing, so that process is not very unified. *(P2-physiotherapist)*

		• I think the surgeons are probably time poor in terms of them necessarily have the opportunity to have those discussions *(P10 – anaesthetist)*<br>• We don't necessarily have the time resources to be getting every single one of these patients in for a one-on-one assessment where we go through extensive screening of this person *(P23 – physiotherapist)*
		**5. System-related implementation factors**
**5.1**	Timing of prehabilitation relative to surgery is challenging due to unpredictable surgery dates and prolonged waitlists (B)	• After 3 months of waiting and doing the prehab, to be told I was probably looking at another 6 to 12 months until surgery, I just fell into a bit of a hole. I felt like I gave up. I've done everything...It's not fair. I don't think it's fair on anyone... you kind of go "well what's the point?" That's why I sort of gave up on my prehab strengthening and all that because I didn't have an end game. *(P7 – patient, male, 56, post-THA)*<br>• The only thing that I want to know is when eventually is it going to happen? I haven't heard anything. I am happy to do whatever they think will help me prepare but it's hard to stay motivated without an end goal in mind, you know? *(P27 – patient, male, 75, pre-THA, CALD background)*<br>• It seems a little bit random when people come in, like it's ideally sort of trying to be within a few months of the surgery, but surgery dates change. Some people come in and they've only got sort of a few weeks before they have the surgery and other people, they don't have a surgery date yet set, so they're coming in and it's very uncertain, like is this the right time to do the exercise? *(P1 – physiotherapist)*<br>• The reason I don't get involved as much is cause of the time frame. There's no point writing out all these referrals if they're just gonna go in a pile and you don't know exactly when they're gonna get their surgery. *(P19 – surgeon)*<br>• It's being able to deliver the right information at the right time that we currently are not able to do. *(P23 – physiotherapist)*<br>• Especially info on you know how long you're waiting for an operation, it really affects you not knowing that. It makes it hard to plan, and set goals. *(P13 – patient, female, 66, pre-TKA)*<br>• I think the hard thing for us as surgeons is that once they're on the waiting list, we don't tend to see them until the surgery, and this is a problem. We're not able to monitor their condition. *(P17 – surgeon)*<br>• But I guess I don't really have a way of evaluating whether you know they've...Whether they've improved enough in six weeks of prehab or if for example, they don't get the surgery for another two months, will they continue the exercise? I don't really know what happens after they leave the prehab group…I would change the target of the exercise if I knew for certain the time frame, but not knowing for certain I feel like I end up giving just a blanket approach to more people and that it's not as targeted. *(P1 – physiotherapist)*
**5.2**	The current care pathway lacks communication and monitoring between	• I just felt so helpless you know….That waiting time before the op was really hard mentally. You don't get a lot of information *(P7 – patient, male, 56, post-THA)*

	patient and health service (B)	• The only negative I find with that is the waiting and not getting answers. You're going through a fair bit emotionally and not know a lot of stuff. There was just no communication, it felt really lonely in that time before the operation. *(P16 – patient, male, 71, post-TKA)*<br>• I didn't know what to expect going into the operation. I was a little bit scared. *(P25 – patient, female, 79, post-TKA, CALD background)*<br>• I do think that that liaison between patient and hospital, patient and social workers or doctors or whoever it is that's doing all that is really, really important because it's a scary time for all of the patients. *(P16 – patient, male, 71, post-TKA)*<br>• So there was really no contact, you know only "oh you are still on the waiting list, you still want the operation?" That was the only contact you get. *(P3 – patient, female, 68, post-THA)*<br>• Everybody, family, friends, they all ask you what's happening, what's happening, and you want to tell them but you don't know because that information isn't forthcoming. So I think sometimes that patient relationship of letting them know everything as soon as you can is really, really important. You're dealing with people's lives and emotions and not just the patient, but everybody else. *(P16 – patient, male, 71, post-TKA)*<br>• I think the hard thing for us as surgeons is that once they're on the waiting list, we don't tend to see them until the surgery, and this is a problem. We're not able to monitor their condition. *(P17 – surgeon)*
**5.3**	Access to information and resources in the pre-operative time is desired and valued when received but can be lacking (B/E)	• I mean, I googled, you know, like what to expect and all that kind of thing to find out that information. *(P3 – patient, female, 68, post-THA)*<br>• You don't really get told anything about how to prepare for the surgery. *(P13 – patient, female, 66, pre-TKA)*<br>• I would've liked to know more about what to expect to feel like afterwards. That would have helped me mentally...It feels like you're going in blindly. *(P24 – patient, female, 52, post-THA)*<br>• We have nothing at all that a patient can access about their hospital stay, and my personal feeling that this completely disempowers them and it makes them reliant on us. *(P23 – physiotherapist)*<br>• But we just don't have the physical information to give to patients. *(P21 – surgical liaison nurse)*<br>• We've got access to really good equipment like a gym space and access to the hydro pool. But I know other health care networks might not have that luxury of having the facilities available, or they might have other competing like groups that you know, they just don't have the time or resources to be able to complete the prehab programs. *(P9 - physiotherapist)*
**5.4**	Prehabilitation referral processes are unclear and programs are thought to be difficult to access (B)	• But if I knew of a specific referral pathway then I would probably refer them. But if there's no referral pathway, I probably wouldn't because I don't see any way that I can actually send them to prehab. *(P10 – anaesthetist)*<br>• I think a big barrier that we've come across is receiving the referrals for prehab. Obviously we need to receive the referrals to be able to deliver the prehab. *(P9 – physiotherapist)*<br>• I've found patients who have wanted to do prehab, but then like linking patients into prehab like closer to home can be really challenging. So a lot of community health centres have really long wait lists, so they're probably going to get a

		• surgery date before they even get offered prehab. And a lot of outpatient departments I've found, even within Metro Melbourne, don't offer a prehab service. *(P9 – physiotherapist)*<br>• But broadly speaking, if I put someone on for a replacement, I won't send a referral saying please prehab them. Part of that is maybe a false assumption that it's hard to get into. *(P19 – surgeon)*
**5.5**	Tensions between equitable access and ethical autonomy in prehabilitation (B)	• It probably would have helped me to gain strength before the operation, but there was no one to guide me in how to do that, I wasn't offered it. *(P24 – patient, female, 52, post-THA)*<br>• But we definitely don't get referrals to prehab for all patients undergoing a joint replacement. Only a portion. And it seems really ad hoc who gets referred and who doesn't. I don't really understand it. *(P2 – physiotherapist)*<br>• I feel like everyone should have the equal opportunity to complete prehab. But that's certainly not how it is currently. *(P9 – physiotherapist)*<br>• I would think it's fair and beneficial to the patients. I mean, I don't know if patients can refuse it. *(P21 – surgical liaison nurse)*<br>• For me a big ethical question is do patients have the capacity to refuse prehabilitation? Do they really understand the benefits? Which then puts us in an ethical quandary, maybe we need to it's something you have to do and you can't get the operation without it. *(P20 – anaesthetist)*
**5.6**	There is uncertainty about value and patient selection in the context of scarce resources (B)	• I wonder though, if the downside of providing prehab is more to the health service. Until we know what's valuable, what's evidence based, and what impacts outcomes, you know, is it a good use of resources? *(P11- physiotherapist)*<br>• From a health economics side, are you taking away from something else? Can we use that money elsewhere that's better? *(P18 – surgeon)*<br>• So I find it a really difficult balance with these patients as we currently stand, to give them best practice care, without using every resource that we have. *(P23 – physiotherapist)*<br>• But I'm just also aware you've got limited resources and the question of who do you target is a great one. We need to know who needs it. *(P12 – anaesthetist)*<br>• My gut feel is that we're spending the majority of our time on the people who don't need us. *(P23 – physiotherapist)*<br>• Let's say we do [prehab] with everybody and we have some measure about "goodness", and then there's a cohort that we can identify that we increase on whatever scale, say one's a 10 and another one's only two. Do we put all the resources into that cohort that we only get two out of improvement or do we put it all in the 10? *(P18 – surgeon)*
**5.7**	Pre-operative care should be tailored to individual needs, however it is currently not needs-based (B/E)	• Different people want different information at different times *(P18 – surgeon)*<br>• The less I know, the better. *(P14 – patient, female, 77, pre-TKA)*<br>• And this is something we currently don't do in our model of care...We don't screen for risk and provide a patient-specific prehab service. Prehab should adapt to the person, not the other way around *(P1 – physiotherapist)*<br>• I mean, from my experience of behaviour change, you need time to provide that education and some people need less time and input and some need more. You've got to tailor it. *(P2 – physiotherapist)*

		• What works for one person doesn't always work for another person. You've gotta make it work for the person, it can't be this blanket approach which is kind of how it feels. *(P7 - patient, male, 56, post-THA)*<br>• It's about providing the best service, tailored to what they need. *(P1 – physiotherapist)*<br>• My gut feel is that we're spending the majority of our time on the people who don't need us. *(P23 – physiotherapist)*
**5.8**	Access to multi-disciplinary team members is desired but difficult (B)	• Well, I just think [the physiotherapists] helped me so much talking to me, explaining to me about the exercises, why you're doing this one and that sort of thing. I mean, you can give people a page full of exercises and they can do it, but they don't know what they're doing it for. *(P6 – patient, female, 72, post-THA)*<br>• Yeah maybe having someone you could reach out to talk to? Someone who you can just discuss pre-op what you're going through mentally. There wasn't that help. Yeah, there was not that help. *(P7 – patient, male, 56, post-THA)*<br>• From my experience in the pre admission setting, there's been no access to get these patients to psych, or dietitians for weight loss, to prepare them for surgery. *(P11 – physiotherapist)*<br>• At the moment the burden sits solely with us in physio and that's a really challenging thing when we don't have the resources to refer to dietetics and to psychology….we've really had to narrow it to focus just on exercise because we haven't had funding for anything else. *(P23 – physiotherapist)*

= Voiced by both healthcare professionals and patients

= Voiced by healthcare professionals only

= Voiced by patients only

Abbreviations: B/E = combined barrier and enabler; B= barrier; E= enabler; TDF: Theoretical Domains Framework; TFA: Theoretical Framework of Acceptability; CALD = culturally and linguistically diverse

eTable 7. Sub-themes and example quotes for Concept 3: 'Strategies for successful implementation of prehabilitation'

Sub-themes	Example quotes
**What should prehabilitation involve?**	
**Empower with education**	• I think the education session could nearly be, you know, one or two sessions in that period of time, whether that time be six weeks. But I feel like that education could be spread out and then provided with follow up online for them to read in their language. *(P4 – physiotherapist)*<br>• I would've liked more information about nutrition definitely, you know, a healthier diet. Maybe like I said, I had a bit of a drinking problem, maybe there could be a thing in there about that as well. I'd say that's part of nutrition. Just about that would've been good. *(P7 – patient, male, 56, post-THA)*<br>• I think just having that advice and knowledge on the benefits would actually get me in the right frame of mind to do things. *(P8 – patient, male, 72, post-TKA*)<br>• I don't know whether the resources are there or the time factors, but it would be great to have somebody sit down with you and go through things, tell you everything's going to be fine, what you can expect afterwards, how to prepare mentally, all of that…and then give you some information to take home and read. I would have jumped at that. *(P16 – patient, male, 71, post-TKA)*<br>• Just advice from professionals on how to deal with sitting around... and in all aspects really. On the exercise, on what sort of medication to take, what you shouldn't take, diet, things like that. Would have been a help instead of having to find out yourself, having to look up and do everything yourself because doesn't mean to say everything you read is correct either. *(P13 – patient, female, 66, pre-TKA)*
**Peer support**	• When everyone comes in the same boat and you can, you can kind of get people at different stages and you can get that information from, you know, someone that's just like you, that has got things through just like you and they've come out the other end. That is really powerful. *(P5 – patient, female, 67, post-THA)*<br>• I would suggest having a session where a number of people get together and explain what the benefits are and what your issues are, and you can learn from each other. *(P8 – patient, male, 72, post-TKA)*<br>• So I think the group class environment is really valuable because you know it's all good and well if we tell them something. But it's even more powerful if they discuss it amongst each other and so they can bounce ideas off each other, they can learn off each other, and say this worked for me – it's probably even more powerful than me telling them what you need to exercise because this and that. *(P15 – physiotherapist)*

**Clearly defined goals**	• I think you have to limit the definition to the end of the preoperative phase because then anything could happen after that which is not the fault of prehabilitation right? So a very simplistic approach to defining a successful prehabilitation would be to say you make the patient as good as they can be before the operation. *(P20 – anaesthetist)*<br>• But it's all messy cause the pathway is so unclear. We need to differentiate and have really clear goals and objectives of prehab and conservative management. *(P23 – physiotherapist)*
**When should prehabilitation be delivered?**	
**Throughout the waitlist journey**	• I think if we're identifying someone needs surgery and they're quite deconditioned and I think that's probably the best time to address it, even if you know their surgery time may be well over a year. They may need a bout then and then another lot of prehab closer to the time. *(P4 – physiotherapist)*<br>• Probably we need to do a 2-speed thing, so you get put on [the waitlist] get given the one pager with 'this is what you can do for yourself'. Maybe contact them again three monthly. And then you kick it up a gear closer to surgery, that's where you get them to come in and do a supervised program. *(P18 – surgeon)*<br>• Certainly I would want information over a period of time. So not just that once, not just right when you're gonna go in for the op[eration]. *(P16 – patient, male, 71, post-TKA)*
**Responsive and adaptive**	• I think there should be more communication between the patients and system, just to check in and keep you updated. Because it's a scary time for all of the patients. *(P16 – patient, male, 71, post-TKA)*<br>• I think they should make more contact with the patient, even just like a phone call to see actually how you are going and if things have got any worse. And have you got problems mentally anything you know, sort of thing I think would be a great help. *(P13 – patient, female, 66, pre-TKA)*<br>• I think we need to be monitoring [patients] whilst they're on the waitlist, and I think there is an enormous opportunity here for the utilisation of [patient reported outcome measures] to monitor significant patient deterioration, and then respond accordingly. *(P23 – physiotherapist)*
**Who should be targeted and/or involved in prehabilitation?**	
**Tailored to the individual**	• It's about providing the best service, tailored to what they need. *(P1 - physiotherapist)*<br>• I mean, just from my experience, for behaviour change you know you need to provide that education and some people need less, some need more. So assessment needs to be individualised. *(P2 – physiotherapist)*<br>• Prehab should be really individualised. So it's like a targeted therapy, set at the right intensity, and appropriately progressed. What works for one person doesn't always work for another. *(P7 – patient, male, 56, post-THA)*

**Multi-disciplinary approach**	• It would have been great to hear from a dietician...It would be very helpful to do something like that. *(P6 – patient, female, 72, post-THA)*<br>• In an ideal world, it would be multidisciplinary, so obviously optimising someone physically is one part, but also optimising patients from like a dietetics and nutrition point of view I also think it's really important. Another thing I feel like would be really helpful is for patients who do have high levels of anxiety having some sort of like psych input preoperatively would be extremely beneficial. *(P9 – physiotherapist)*<br>• So my feeling is prehab would be that concept of a whole package of things that gets people ready…there should be a team including dietetics, psychology, maybe pain specialists, physio…there need to a be a dedicated multidisciplinary clinic that we can access. *(P18 – surgeon)*
**Coordinated care team**	• It would be good to have a person that kind of coordinates everything and has access to the surgical team. They need to be an expert across multiple fields, I'd say. *(P2 – physiotherapist)*<br>• I think the message needs to be consistent across the board…everyone in the team, the message needs to be the same. *(P1 - physiotherapist)*
**How should prehabilitation be delivered?**	
**Multi-modal delivery**	• I feel if we could have [prehab] locally to them in a good time frame, then we'd get so many more people doing it. *(P4 - physiotherapist)*<br>• You could provide the education in lots of multimedia, so you can provide video, you can provide online content written content in different like translated in all languages. (P10 - *anaesthetist*)<br>• I'm a big believer in in-person…But if you could give me a program and every week I touch base on telehealth and say 'yes, I've done my program this week' instead of having to come all the way here. That's an option. *(P7 – patient, male, 56, post-THA)*
**Operational efficiency**	• The more laborious the referral is that that's going to be a barrier.... It should be just somehow automated *(P10 – anaesthetist)*<br>• So you don't want somebody have to go, "Oh, yeah. I've got to remember to do this" or whatever. Even having reminders...to remember to refer these patients to prehab, so have posters that can also be. *(P18 – surgeon)*

References

1. Cane J, O'Connor D and Michie S. Validation of the theoretical domains framework for use in behaviour change and implementation research. *Implement Sci* 2012; 7: 37. DOI: 10.1186/1748-5908-7-37.
2. Sekhon M, Cartwright M and Francis JJ. Acceptability of healthcare interventions: an overview of reviews and development of a theoretical framework. *BMC Health Serv Res* 2017; 17: 88. 20170126. DOI: 10.1186/s12913-017-2031-8.
3. Paynter C, McDonald C, Story D and Francis JJ. Application of the theoretical framework of acceptability in a surgical setting: Theoretical and methodological insights. *Br J Health Psychol* 2023; 28: 1153-1168. 20230624. DOI: 10.1111/bjhp.12677.
4. Tong A, Sainsbury P and Craig J. Consolidated criteria for reporting qualitative research (COREQ): a 32-item checklist for interviews and focus groups. *Int J Qual Health Care* 2007; 19: 349-357. 20070914. DOI: 10.1093/intqhc/mzm042.
